# Claspin recruits Cdc7 kinase for initiation of DNA replication in human cells

**DOI:** 10.1038/ncomms12135

**Published:** 2016-07-12

**Authors:** Chi-Chun Yang, Masahiro Suzuki, Shiori Yamakawa, Syuzi Uno, Ai Ishii, Satoshi Yamazaki, Rino Fukatsu, Ryo Fujisawa, Kenji Sakimura, Toshiki Tsurimoto, Hisao Masai

**Affiliations:** 1 Department of Genome Medicine, Tokyo Metropolitan Institute of Medical Science, 4-6-1 Kamikitazawa, Setagaya-ku, Tokyo 156-8506, Japan; 2 Department of Biology, Faculty of Science, Kyushu University 744 Motooka, Nishi-ku, Fukuoka 819-0395, Japan; 3 Department of Cellular Neurobiology, Brain Research Institute, Niigata University, Niigata 951-8585, Japan

## Abstract

Claspin transmits replication stress signal from ATR to Chk1 effector kinase as a mediator. It also plays a role in efficient replication fork progression during normal growth. Here we have generated conditional knockout of *Claspin* and show that *Claspin* knockout mice are dead by E12.5 and *Claspin* knockout mouse embryonic fibroblast (MEF) cells show defect in S phase. Using the mutant cell lines, we report the crucial roles of the acidic patch (AP) near the C terminus of Claspin in initiation of DNA replication. Cdc7 kinase binds to AP and this binding is required for phosphorylation of Mcm. AP is involved also in intramolecular interaction with a N-terminal segment, masking the DNA-binding domain and a newly identified PIP motif, and Cdc7-mediated phosphorylation reduces the intramolecular interaction. Our results suggest a new role of Claspin in initiation of DNA replication during normal S phase through the recruitment of Cdc7 that facilitates phosphorylation of Mcm proteins.

## Abstract (web summary)

Claspin mediates the transmission of a replication-stress signal from ATR to Chk1 and is necessary for efficient fork progression. Here the authors demonstrate that the C-terminal acidic patch is important for this role due to its interaction with Cdc7.

## Graphical abstract

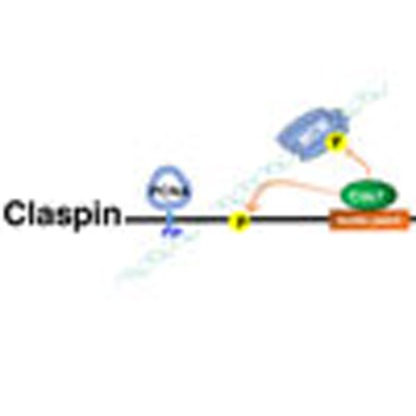

Claspin was originally discovered as a factor that binds to Chk1 and is essential for activation of Chk1 in *Xenopus* egg extract^1^. Phosphopeptide motifs were discovered on Claspin that are required for regulated binding of Chk1 (ref. 2). Subsequently, human Claspin was also shown to be required for replication checkpoint control^3^,^4^,^5^. Claspin is loaded onto chromatin in a manner dependent on pre-RC and Cdc45, but not on RPA in *Xenopus* egg extracts^6^. Biochemically, Claspin is a ring-like structure with DNA-binding activity with some preference for forked structures^7^,^8^.

During the normal course of DNA replication, Claspin is required for efficient fork progression^9^,^10^,^11^. This feature appears to be conserved also in budding yeast Mrc1, yeast homologue of Claspin^12^. Claspin interacts with various replication factors including ATR, Chk1, Cdc7 kinase, Cdc45, Tim, MCM4, MCM10, PCNA, DNA polymerases α, δ, ɛ and And-1 (refs 8, 13, 14, 15, 16), suggesting its role at the replication forks and potentially in initiation. Yeast Mrc1 was shown to move along with replication fork, linking the helicase components to the replicative polymerases^17^.

We previously reported that Claspin is phosphorylated in a manner dependent on Cdc7 kinase^18^. Subsequently, Cdc7-dependent phosphorylation of Claspin was shown to be required for Clspin–Mcm2 interaction^19^. In *Xenopus* egg extract, interaction between Claspin and Drf1/ASKL1, a second activation subunit for Cdc7 kinase, was reported^14^. In fission yeast, Hsk1 (Cdc7 homologue) interacts with and phosphorylates Mrc1 (ref. 20). Thus, physical and functional interactions between Cdc7 and Claspin/Mrc1 may be conserved.

Whereas roles of Claspin in replication checkpoint control have been studied intensively, those in normal replication have been largely unclear. In this report, to clarify the roles of Claspin in regulation of normal DNA replication, we constructed genetically engineered mice and cells in which Claspin could be inducibly knocked out. Using the mutant cells, we have identified C-terminal acidic patch sequence that is essential for non-checkpoint functions of Claspin. The acidic patch is required for Claspin to bind to Cdc7 kinase and to be phosphorylated by this kinase. It interacts also with a N-terminal segment containing DNA-binding domain and the newly identified PIP (PCNA-interacting protein) motif and suppresses DNA and PCNA bindings. Cdc7 is recruited to the acidic patch and phosphorylates Claspin, which leads to reduced interaction between the acidic patch and the N-terminal segment. This in turn would lead to increased DNA and PCNA bindings. More importantly, the DE/A mutant in which all the acidic residues were replaced by alanine, did not interact with Cdc7 and exhibited both growth and BrdU incorporation defects in mouse embryonic fibroblast cells. Cdc7-mediated phosphorylation of critical residues on Mcm was specifically reduced with the DE/A mutant in these cells. These results suggest that Claspin plays an important role in recruiting Cdc7 kinase most likely for efficient initiation of DNA replication in normal mammalian cells.

We report here that an acidic patch present near the C terminus of Claspin interacts with Cdc7. It also interacts with a N terminus proximal segment that contains a DNA-binding domain and a PCNA-binding PIP motif, causing most likely intramolecular looping. We further show that the acidic patch plays dual roles for the processes of DNA replication through interaction with Cdc7. First, Cdc7 kinase recruited to the acidic patch facilitates phosphorylation of Mcm required for initiation of DNA replication. Second, it promotes DNA and PCNA bindings of Claspin through inhibiting its intramolecular interaction.

## Results

### Generation of *Claspin* knockout mutant mice and MEF cells

**Figure 1:**
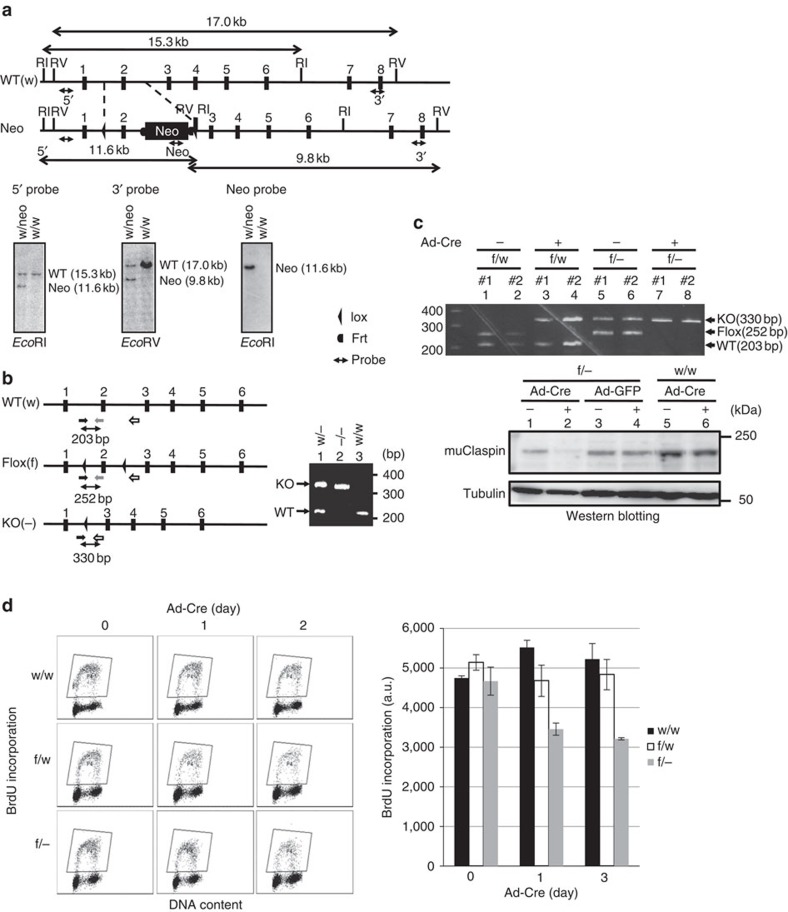
Generation of *Claspin*-deficient mice. (**a**) Upper drawing, schematic representation of wild-type, flox and knockout alleles of mouse *Claspin.* RI: *Eco*RI site. EV: *Eco*RV site. Lower panels, Southern blot analysis of genomic DNA from the wild-type ES clone (w/w) and G418-resistant *Claspin*^+/−^ heterozygous ES clone (w/neo). Neo, neomycin-resistance gene. (**b**) Characterization of *Claspin* knockout embryos. Upper drawing, maps showing the locations of primers (shown by arrows; black, Cg1F; grey, Cg1mR; white, Cg2R) used for genotyping of mutant mice. Lower, a table showing the numbers of embryos of each genotype obtained from crosses of heterozygous mice. The right panel shows the genotyping by PCR on genomic DNA isolated from heterozygous (w/−), knockout (KO; −/−) or wild-type (WT; w/w) cells. In **a** and **b** the numbered vertical bars indicate the exons. (**c**) Generation of *Claspin* knockout MEF cells. Cre-loxP induced knockout of *Claspin*. Upper panel, PCR analysis of genomic DNA of MEF cells (f/w and f/−) non-treated (−) or infected with Ad-Cre (+) for 48 h. Lower panels, MEF cells with indicated genotype was infected with Ad-GFP or Ad-Cre for 48 h, and whole-cell extracts were analysed by western blotting to detect the proteins indicated. (**d**) BrdU incorporation in *Claspin* knockout MEF cells. MEF cells (w/w, f/w and f/−) were infected with Ad-Cre and incubated for 0, 1 and 2 days. Before harvest, BrdU was added for 20 min. Left, BrdU incorporation was analysed by FACS. Right, quantification of the result. An average FITC intensity of each cell population is shown. The mean±s.d. values from three replicates are shown.

To genetically dissect the functions of Claspin in development and in cell proliferation, we have generated conditional knockout mice. LoxP sequences were introduced in the introns before and after the second exon (Fig. 1a). The expression of Cre recombinase results in deletion of the second exon containing the initiation codon, leading to inactivation of *Claspin*. Whereas crossing of +/− and +/− mice generated −/− embryos at E9.5, albeit at a rate lower than expected, no −/− embryos were detected at E12.5, suggesting that *Claspin* knockout mice is non-viable by E12.5 (Fig. 1b; Table 1). We have generated flox/− (f/−) mouse embryonic fibroblast (MEF) cells, and infected them with recombinant adenovirus encoding Cre recombinase (Ad-Cre), which resulted in loss of Claspin expression (Fig. 1c). We noted that growth was retarded and DNA synthesis, as measured by BrdU incorporation, was also reduced upon Ad-Cre infection (Fig. 1d). This is consistent with previous report on cancer cells depleted of Claspin by siRNA^9^,^10^. Hydroxyurea-induced Chk1 activation (phosphorylation of S345) was also reduced in Ad-Cre-infected *Claspin*^f/−^ MEF cells, as was previously reported for Claspin-depleted cancer cells^9^ (Supplementary Fig. 1).

### Claspin binds to replication factors through an acidic patch

**Figure 2:**
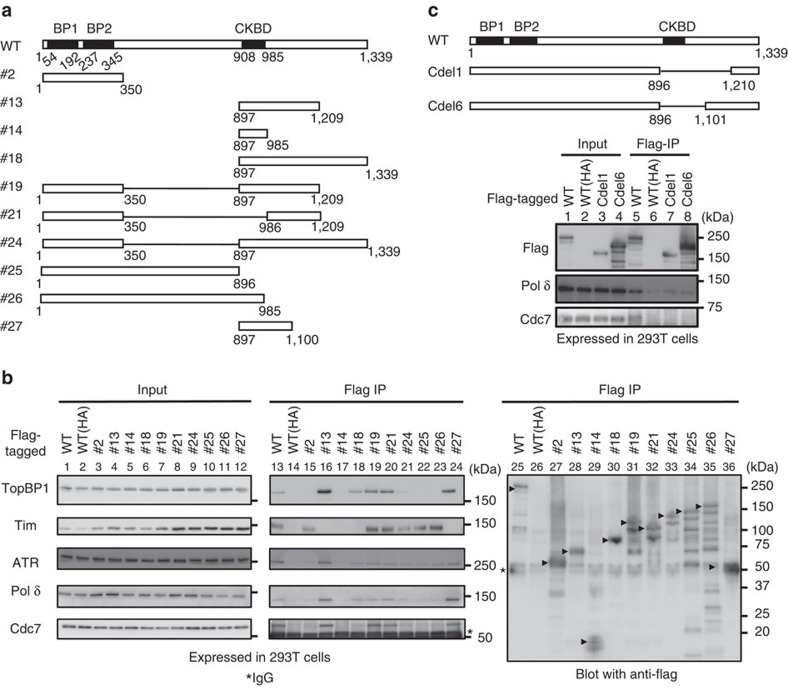
Interaction of various replication factors with truncated or mutant forms of Claspin. (**a**) Schematic diagram of various truncated or mutant derivatives of Claspin generated. BP1 and BP2: basic patch1 and 2. CKBD: Chk1 binding domain. (**b**) Mutant Claspin polypeptides tagged with 3x Flag at the C termini were expressed in 293T cells and pulled down with M2 Flag beads (Flag IP). Co-pulled down proteins were analysed by western blotting to detect the proteins indicated. Asterisk, IgG; black arrowheads, full-length or mutant Claspin polypeptides pulled down by Flag antibody. (**c**) Loss of interaction with Cdc7 in Cdel1 and Cdel6 mutants, whose structures are shown in the upper drawing. Interaction was examined as in **b**.

**Figure 3:**
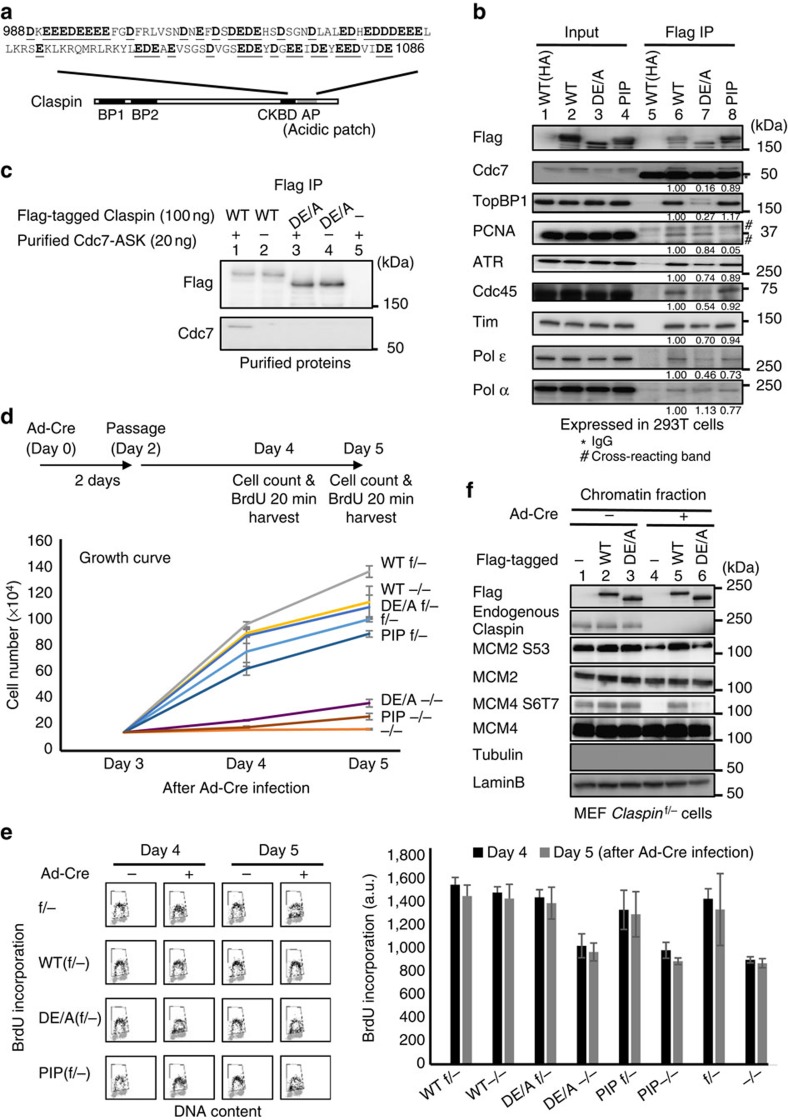
The C-terminal acidic patch is crucial for Claspin functions. (**a**) Amino-acid sequences of aa988–1,086 (acidic patch, grey box) of Claspin. Acidic amino acids are underlined. In DE/A mutant, all these residues were replaced with alanine. (**b**) Wild-type (WT) and mutant *Claspin* indicated were expressed in 293T cells and were pulled down with M2 Flag beads. Associated proteins were analysed by western blotting to detect the proteins indicated. Values under each panel in lanes 6, 7 and 8 are the amount of each coimmunoprecipitated protein in Flag IP relative to that of the input protein. The values for the wild-type Claspin are taken as 1.00. (**c**) Purified wild-type or DE/A mutant Claspin was mixed with purified Cdc7-ASK complex and pulled down by Dynabeads conjugated with anti-Flag antibody. Immunoprecipated materials were analysed by western blotting, as shown. (**d**) Stable clones of f/− MEF cells expressing WT, DE/A and PIP were infected with Ad-Cre (+) or non-treated (−) for 2 days and 1.0 × 10^5^ cells were passaged to new plates. The cells were then harvested at 2 days (Day 4 from Ad-Cre infection) and 3 days (Day 5 from Ad-Cre infection) after the passage and cell numbers were counted. The drawing shows the scheme of experiments. The mean±s.d. values from three replicates are shown. (**e**) Quantification of the BrdU incorporation of cells in **d**. Before harvest, BrdU was added for 20 min, and BrdU incorporation was analysed by FACS. The average FITC intensity of each cell population is shown in the graph. The mean±s.d. values from three replicates are shown. (**f**) Chromatin-enriched fractions of Ad-Cre treated or non-treated Claspin MEF (f/−) stably expressing the wild-type or DE/A mutant Claspin were analysed by western blotting to indicate proteins indicated and their phosphorylated forms.

We and others previously reported that Claspin interacts with many factors involved in checkpoint regulation and replication fork machinery, including ATR, ATM, Chk1, Tim, MCM4, MCM10, Cdc45, DNA polymerases α, δ, ɛ, And-1 and Cdc7 kinase^8^,^13^,^14^,^15^,^16^,^18^. We have generated a series of deletion derivatives of Claspin tagged with Flag at its C terminus (Fig. 2a), transiently expressed them in 293T cells and examined their interaction with these factors by immunoprecipitation using anti-Flag antibody (Fig. 2b). We noted that interaction of Claspin with Cdc7, TopBP1 and Pol δ largely depends on the presence of a C-terminal segment. The presence of this C-terminal segment similarly facilitated the interaction with ATR, but weak binding to ATR was observed with #25 (aa1–896) or #26 (aa1–985) (Fig. 2b, lanes 22 and 23). The 204 amino-acid segment (#27, aa897–1,100) was sufficient for binding to Cdc7, TopBP1 and Pol δ (Fig. 2b, lane 24), while #14 (aa897–985) did not bind to any proteins examined (Fig. 2b, lane 17), suggesting that aa986–1,100 is essential for the binding. On the other hand, the internal deletion of 897–1,209 or 897–1,100 resulted in complete loss of binding to Cdc7 as well as in significantly reduced binding to Pol δ (Fig. 2c, lanes 5, 7 and 8). This segment (aa986–1,100) is highly enriched in acidic residues (aspartic acid and glutamic acid; see Fig. 3a), and hereafter will be designated AP (acidic patch).

We also noted that #18 (aa897–1,339) and #24 (aa1–350+aa897–1,339) bound to Cdc7 or other factors only inefficiently in spite of the fact they contain the acidic region (Fig. 2b, lanes 18 and 21). aa1,210–1,339 is highly enriched in basic amino acids (26 lysines and arginines out of 130) and therefore this region may interfere with the binding of factors to the AP segment. In contrast, the N-terminal segments were required for binding to PCNA and Tim as well as for DNA-binding. In fact, aa1–350 (#2) was sufficient for binding to PCNA and Tim (Fig. 2b, lane 15; see also Fig. 5c).

### The AP is crucial for Claspin functions

To understand the physiological functions of the AP, we constructed a DE/A mutant in which all the aspartic acids and glutamic acids in the AP were substituted with alanine (aa988–1,086; Fig. 3a). We examined the interaction of the DE/A mutant with various proteins after its overexpression in 293T cells, as above. The DE/A mutant did not interact with Cdc7, while it interacted with PCNA, ATR, DNA polymerase α and Tim. Interaction with ATR and Tim was slightly decreased and that with TopBP1, Cdc45 and Pol ɛ was significantly reduced compared to the wild type (Fig. 3b, lane 7). Purified full-length Claspin interacted with purified Cdc7-ASK (Activator of S phase; huDbf4), while the mutant DE/A Claspin did not (Fig. 3c), showing that Claspin directly interacts with Cdc7-ASK. These results support the idea that the acidic amino acids in AP play crucial roles in binding of Claspin to Cdc7 and possibly other replication factors.

We then examined the physiological functions of the DE/A mutant using *Claspin* knockout MEF cells. We have generated retroviruses encoding mutant *Claspin* which had been fused with mAG (monomeric Azami Green fluorescent protein)^21^ at the N terminus. The *Claspin*^f/−^ MEF cells were transfected with the retroviruses and mAG-positive stable cell lines were isolated. Upon Ad-Cre infection, endogenous *Claspin* was knocked out, and growth and DNA replication were examined. The DE/A mutant could not restore the growth, while the wild-type *Claspin* could fully recover the growth of *Claspin* knockout MEF cells (Fig. 3d). The DE/A mutant could not correct the defect of DNA synthesis, either (Fig. 3e). This indicates that the acidic patch is required for Claspin to support normal DNA replication.

### AP is required for phosphorylation of Mcm proteins by Cdc7

Mcms are known to be critical targets of Cdc7 kinase in initiation of DNA replication^22^. Therefore, we examined whether Mcm phosphorylation is affected in *Claspin* knockout MEF cells. We monitored phosphorylation of Mcm2 and Mmc4, using phospho-specific antibodies (Mcm2 S53 and Mcm4 S6T7) that recognize residues known to be phosphorylated by Cdc7 (refs 23, 24). In *Claspin*^−/−^ MEF cells, both Mcm2 S53 and Mcm4 S6T7 signals were significantly reduced (Fig. 3f, lane 4). Similar reduction of Mcm phosphorylation was observed also in NHDF (normal human dermal fibroblast cells) depleted of Claspin by siRNA (Supplementary Fig. 2a, lane 6 and Supplementary Fig. 2b, lanes 3 and 6). Interestingly, the Mcm phosphorylation was not affected in cancer cells (U2OS and HeLa cells) similarly depleted of Claspin (Supplementary Fig. 2a, lane 8 and Supplementary Fig. 2b, lanes 9 and 12), consistent with previous report^25^. We then examined whether the acidic patch of Claspin is involved in Mcm phosphorylation. Phosphorylation of both Mcm2 and Mcm4 was fully restored by ectopic expression of the wild-type full-length Claspin fused with mAG (Fig. 3f, lane 5). In contrast, the DE/A mutant could not restore the phosphorylation of Mcms (Fig. 3f, lane 6). These results strongly suggest that Claspin plays a crucial role in recruiting Cdc7 kinase for phosphorylation of pre-RC through its AP. Interestingly, this requirement for Claspin is specific to normal cells, and cancer cells appear to have acquired mechanisms that bypass this pathway.

### ΔAP or DE/A mutant shows higher DNA-binding activity

**Figure 4:**
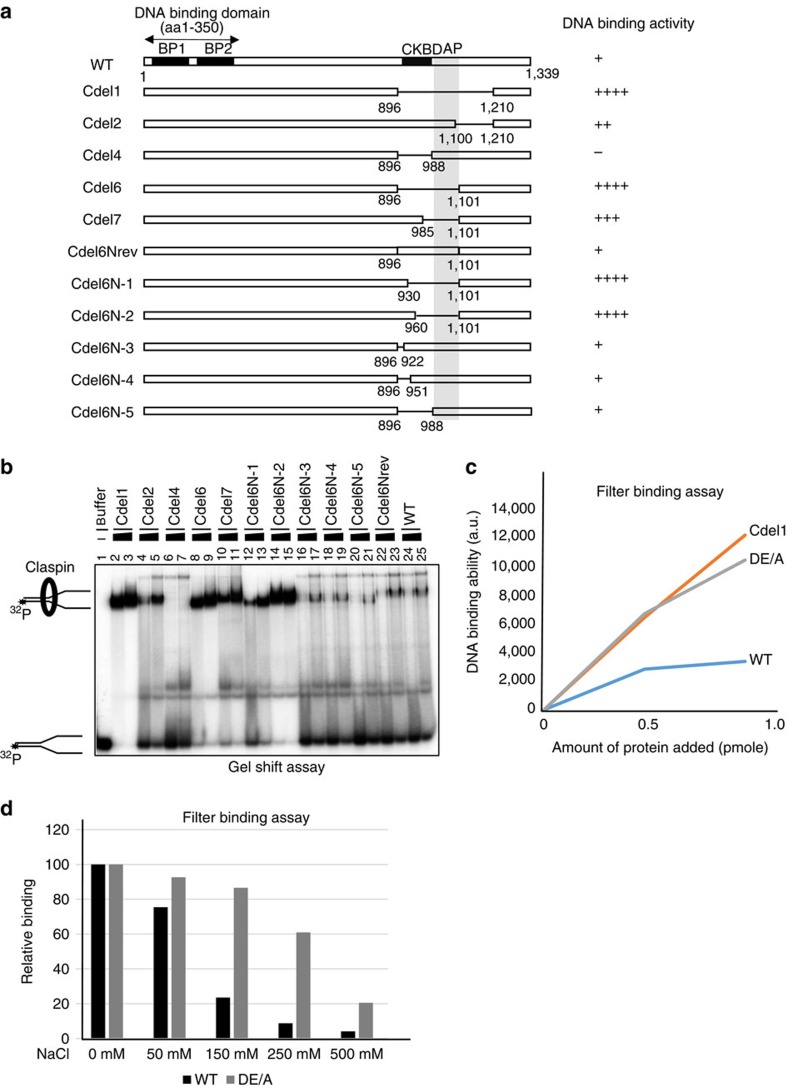
DNA-binding activity of Claspin is regulated by the C-terminal acidic patch. (**a**) Schematic diagram of mutant Claspin-containing internal deletions within the C-terminal segment.+ Marks indicate the relative strength of DNA binding. BP1 and BP2, Basic patch1 and 2; CKBD, Chk1 binding domain; AP, acidic patch. The double arrowed segment (aa1–350) can bind to DNA with high affinity. (**b**) Gel shift assays of mutant Claspin proteins on Y-fork DNA. Y-fork DNA, 20 fmol; protein added, 0.5 and 1.0 pmol. The reason why Cdel4 fails to generate the larger protein–DNA complex is not clear. (**c**) Filter-binding assays of Cdel1 and DE/A mutant Claspin proteins on Y-fork DNA. Y-fork DNA, 80 fmol; protein added, 0.5 and 1.0 pmol. (**d**) Effect of salt on DNA binding activity of full-length and DE/A mutant Claspin proteins. Filter-binding assays were conducted as in **c** using the full-length and DE/A mutant Clapsin proteins (2 pmol), except that filters were further washed by binding buffer containing increased concentration of NaCl. Binding without additional wash was taken as 100.

It was previously reported that Claspin can bind to DNA. It binds to dsDNA but prefers fork-like DNA^7^. We generated various deletion derivatives of Claspin (Fig. 4a), purified them (Supplementary Fig. 3) and assayed their DNA-binding activity in gel shift assays using Y-fork DNA as a substrate (Fig. 4b). DNA-binding activity is lost in mutants lacking N-terminal segments, confirming that DNA-binding activity lies in the N-terminal segment^7^,^8^,^15^,^26^. We noted that derivatives lacking C-terminal domains bind to DNA with much higher affinity than that of the full-length Claspin. The segment responsible for the suppression of DNA-binding was mapped to the AP (aa986 to aa1101) discussed above (Fig. 4b, Cdel1, Cdel6, Cdel7, Cdel6N-1 and Cdel6N-2). Cdel2, lacking 1,101–1,209, showed higher binding activity than the full-length, suggesting that this segment is also involved in the suppression.

We then examined the DNA-binding activity of the DE/A mutant. The DE/A mutant, like Cdel1, showed significantly higher affinity to DNA than the wild-type protein did (Fig. 4c; filter-binding assays). In the similar filter-binding assays, the DE/A mutant showed higher resistance to salt compared with the wild-type protein. At 150 mM KCl, more than 60% binding remained with DE/A, whereas only 20% remained with the wild-type. At 250 mM KCl, 60% remained with DE/A, while only <10% remained with the wild type (Fig. 4d). Furthermore, the DE/A mutant protein, expressed in 293T cells, was enriched in the Triton-insoluble fractions at a low salt concentration (Supplementary Fig. 4). Washing the triton-insoluble pellet with increasing salt indicated that the full-length Claspin dissociated from the pellet at 100 mM NaCl, whereas a portion of the DE/A mutant Claspin stayed in the pellet even at 400 mM NaCl (Supplementary Fig. 5), indicating higher and salt-resistant affinity of the DE/A mutant to chromatin. These results are consistent with the fact that the DE/A mutant binds to DNA with higher and salt-resistant affinity and suggest that the acidic residues in AP somehow inhibit the DNA-binding activity of the N-terminal DNA-binding segment. Glycerol gradient analyses of the purified DE/A protein showed that the mutant protein migrates at fractions similar to those of the wild type (at a monomer position; Supplementary Fig. 6), excluding the possibility that defects of the DE/A mutant are caused by changes of the overall shape and structure of the protein molecule. The slight tailing of the mutant to higher molecular weight range may reflect its elongated shape due to the loss of intramolecular interaction (see below).

### PIP-dependent interaction of Claspin with PCNA

**Figure 5:**
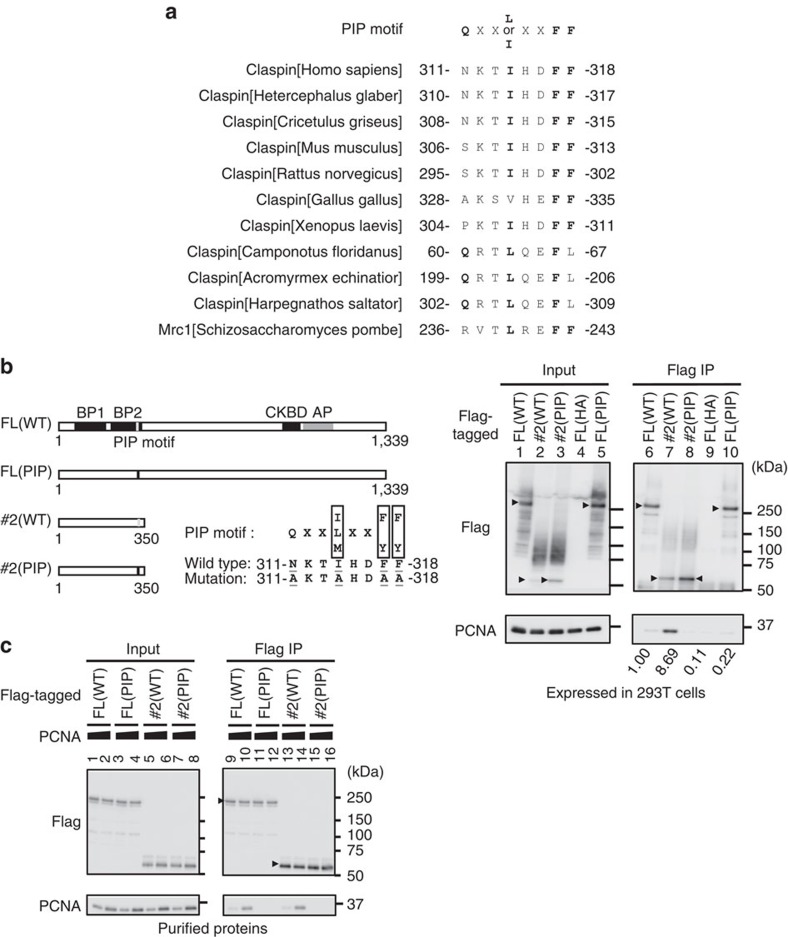
PIP-dependent binding of PCNA to Claspin. (**a**) Comparison of the putative PIP motif sequences of Claspin homologues from various species. Conserved amino acids are in bold. (**b**) Left, a schematic diagram of the full-length and #2 polypeptide of Claspin. Grey and black vertical bars indicate the wild-type and mutant PIP, respectively. Right panels, the wild-type (WT) and PIP mutant (PIP) proteins expressed in 293T cells were pulled down with M2 Flag beads, and analysed by western blotting using anti-Flag or anti-PCNA antibody. FL(HA), HA-tagged wild-type Claspin as a negative control. Arrowheads indicate the full-length and #2 Claspin polypeptides. Values under lanes 6, 7, 8 and 10 represent relative intensities of the coimmunoprecipitated PCNA bands. (**c**) Interaction between purified Claspin polypeptides and PCNA. PCNA, 1.2 or 3.0 pmol; full-length or #2 Claspin polypeptide, 1.2 pmol each. Pulled down materials by Dynabeads-conjugated anti-Flag antibody were analysed by western blotting using anti-Flag or anti-PCNA antibody.

Claspin was reported to interact with PCNA^27^. We have identified a potential PIP motif at aa311–318 (NKTIHDFF) and mutated the conserved residues to alanine (AKTAHDAA, Fig. 5a,b). Both full-length and the N-terminal polypeptide #2 (aa1–350) expressed in 239T cells bound to PCNA in immunoprecipitation (Fig. 5b, lanes 6 and 7). We noted that the polypeptide #2 consistently bound significantly more PCNA compared with the full length. In the same assay, the PIP motif mutants generated on the full length and #2 did not bind to PCNA (Fig. 5b, lanes 8 and 10). The residual binding of the full-length PIP mutant to PCNA, albeit at a low level, may indicate the presence of another PIP-like sequence. The PIP mutant is defective specifically in interaction with PCNA and could interact with other replication factors (Fig. 3b, lane 8). We also examined the interactions using purified proteins. The purified full-length Claspin or #2 polypeptide (aa1–350) were mixed with PCNA and immunoprecipitation by anti-Flag antibody was conducted. The immunoprecipitate with the wild-type form of Claspin contained PCNA but that with the PIP mutant did not (Fig. 5c, compare lanes 9, 10 and 11, 12; 13, 14 and 15, 16), showing that Claspin directly binds to PCNA through PIP.

Using *Claspin* knockout MEF cells, we examined the functions of the PIP mutant. The PIP mutant could not restore the growth and DNA synthesis of *Claspin*^−/−^ cells, while the wild-type Claspin could fully recover them (Fig. 3d,e). This indicates that PCNA binding is required for Claspin to support normal DNA replication.

### AP binds to ClaspinNter and inhibits its DNA/ PCNA bindings

**Figure 6:**
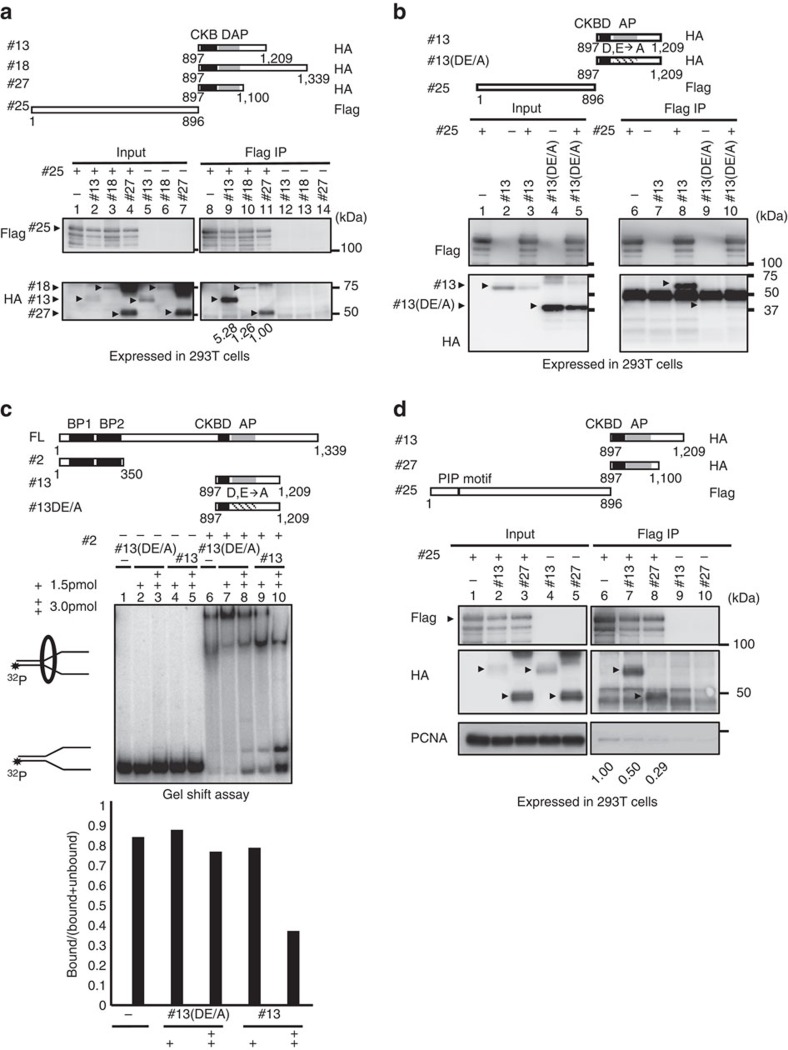
The C-terminal acidic patch interacts with the N-terminal segments of Claspin and inhibits its binding to DNA or PCNA. (**a**) Upper drawing, a schematic diagram of the Claspin polypeptides expressed and their attached tags. Lower, the Flag-tagged N-terminal segment (#25) was coexpressed with the HA-tagged C-terminal polypeptides and pulled down with M2 Flag beads. The inputs and immunoprecipitates (Flag IP) were analysed by western blotting using anti-Flag or anti-HA antibody. Values under the right panel are the amount of the C-terminal polypeptides in Flag IP relative to the input proteins and those for #27 are taken as 1.00. (**b**) The Flag-tagged N-terminal segment (#25) was coexpressed with the wild-type or DE/A mutant C-terminal polypeptide #13 and pulled down with M2 Flag beads, and the inputs and the immunopreipitates (Flag IP) were analysed by western blotting using anti-Flag or anti-HA antibody. Arrowheads indicate the wild-type and DE/A #13 polypeptides. Note that the wild-type #13 polypeptide migrates anomalously on PAGE due to the presence of acidic residues. (**c**) The C-terminal acidic patch polypeptide (#13; 1.5 and 3 pmol) or C-terminal DE/A mutant (#13 DE/A; 1.5 and 3 pmol) was added to the gel shift reaction containing labelled Y-fork (20 fmol) and the N-terminal polypeptide #25 (1.5 pmol). The graph shows the quantification of the binding by calculating the ratio of bound radioactivity to the total radioactivity (bound+unbound). (**d**) The Flag-tagged N-terminal segment (#25) was coexpressed with the HA-tagged C-terminal polypeptides. The inputs and immunoprecipitates (Flag IP) with M2 Flag bead were analysed by western blotting for #25 and associated PCNA. Arrowheads indicate the expressed proteins. Values under lanes 6, 7 and 8 represent relative intensities of the coimmunoprecipitated PCNA bands. Grey box indicate AP and striped box indicate the DE/A mutant AP.

Above results suggest a possibility that the C-terminal segment regulates the interaction of Claspin with DNA and PCNA. To test this idea, C-terminal polypeptides (#13, aa897–1,209; #18, aa897–1,339; #27, aa897–1,100; HA-tagged) were expressed and purified and mixed with the purified N-terminal polypeptide (#25, aa1–896; Flag-tagged). Immunoprecipitates with anti-Flag antibody contained HA-tagged C-terminal polypeptides (Fig. 6a, lanes 9–11), indicating that N-terminal and C-terminal polypeptides of Claspin interact with each other. We noted that the binding of #18 to the N-terminal polypeptide was less efficient than that of #13, a situation similar to the interaction with Cdc7 (Fig. 2b, lanes 16 and 18). The presence of basic residues in aa1,210–1,339 may interfere with the association of AP with the N-terminal segment. #13 bound to #25 more efficiently than #27 did, indicating that the sequence 1,101–1,209 may also be involved in interaction with the N-terminal segment (Fig. 6a, lanes 9 and 11). On the other hand, the #13 polypeptide containing DE/A substitution did not bind to the N-terminal fragment (Fig. 6b, lane 10). Furthermore, addition of the C-terminal polypeptide (#13, aa897–1,209) inhibited the DNA-binding activity of an N-terminal polypeptide (#25, aa1–896; Fig. 6c, lanes 9 and 10), whereas that of the same C-terminal polypeptide containing DE/A mutation in AP did not cause significant inhibition (Fig. 6c, lanes 7 and 8). These results support the idea that the intramolecular interaction between the C-terminal AP and the N-terminal segments containing the DNA-binding domain regulates the DNA-binding activity of Claspin.

Apparent higher affinity of the N-terminal polypeptide to PCNA (Fig. 5b, compare lanes 6 and 7) suggests a possibility that the C-terminal segment may interfere also with the PCNA binding. Consistent with this speculation, expression of the C-terminal polypeptide (#13, aa897–1,209; #27, aa897–1,100) decreased the binding of PCNA to the N-terminal polypeptide (Fig. 6d; compare lane 6 with lanes 7 and 8). Thus, the C-terminal acidic patch inhibits DNA-binding and PCNA-binding activities of Claspin through directly interacting with the N-terminal segment.

### Cdc7 inhibits interaction between AP and N-terminal segment

We previously reported that Cdc7 is required for HU-mediated checkpoint responses and Claspin is phosphorylated in a manner dependent on Cdc7 kinase^18^. Later, use of Cdc7 inhibitors confirmed this finding^19^.

**Figure 7:**
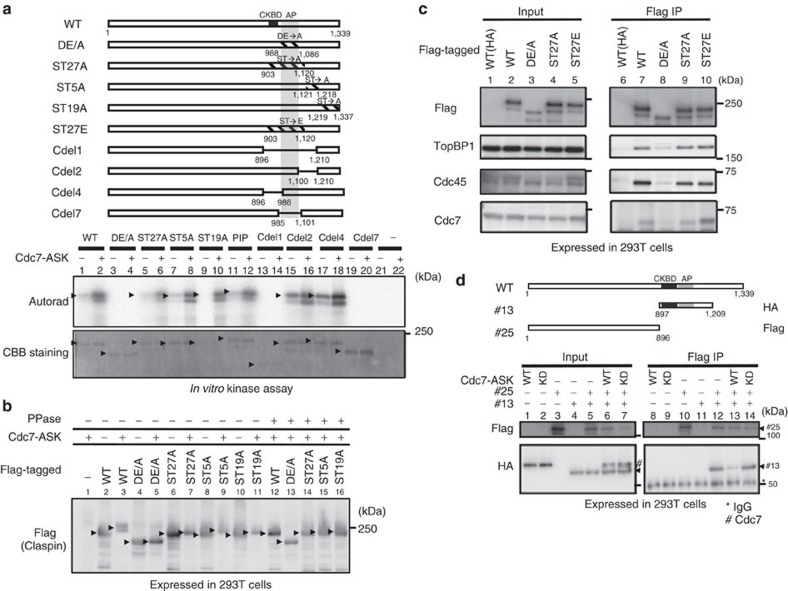
Cdc7 phosphorylates Claspin in a manner dependent on AP and inhibits N–C interaction. (**a**) *In vitro* Cdc7-ASK kinase assays with wild-type and mutant Claspin proteins as substrates. Upper, schematic diagrams of Claspin derivatives including DE/A and other Claspin mutants in which serine/ threonine residues were replaced with alanine or glutamic acids; middle panel, autoradiogram; lower panel, CBB staining. Striped boxes indicate the segments in which serines/threonines were replaced with alanine or glutamic acid. (**b**) The wild-type or mutant Claspin proteins, as indicated, were coexpressed in 293T cells with Cdc7-ASK expression vectors. The whole-cell extracts were analysed by western blotting with anti-Flag antibody. Lanes 12–16, the samples were pretreated with lambda phosphatase before loading. (**c**) Wild-type (WT) and mutant Claspin proteins, as indicated, were expressed in 293T cells and were pulled down with M2 Flag beads. Associated proteins were analysed by western blotting to detect the proteins indicated. WT(HA) represents the HA-tagged wild-type Claspin used as a negative control. (**d**) The Flag-tagged N-terminal segment (#25) was coexpressed with the HA-tagged C-terminal polypeptide (#13) along with HA-tagged Cdc7 (wild-type or kinase dead^28^) and myc-tagged ASK and pulled down with M2 Flag beads. Association of #13 was analysed by western blotting using anti-HA antibody (lower panel). Arrowheads indicate the pulled down #13 polypeptide. *IgG; ^#^Cdc7 polypeptide reacting with anti-HA antibody.

To examine the roles of Cdc7-mediated phosphorylation of Claspin, we tried to localize the phosphorylation sites on Claspin. Since the target sequences of Cdc7 kinase are rather promiscuous except that it favours acidic environment near the phosphorylation sites^23^,^28^,^29^,^30^,^31^, they are generally hard to predict. It is also often the case that multiple residues are phosphorylated by Cdc7 and the phosphorylation of these residues redundantly contributes to the physiological effect^23^. Indeed, nine potential Cdc7-mediated phosphorylation sites *in vitro* have been reported on Claspin^19^. Thus, we have mutated clusters of serine/threonine residues in the C-terminal segment near the acidic patch. ST19A, ST5A and ST27A mutants carry alanine substitutions of all the serine and threonine residues within the segments aa1,219–1,337, aa1,121–1,218 and aa903–1,120, respectively (Fig. 7a). These phosphorylation site mutant Claspin proteins were purified and were used as substrates for *in vitro* phosphorylation assays with Cdc7 kinase. Although ST27A mutant was less efficiently phosphorylated by Cdc7 than the wild type was (Fig. 7a, compare lanes 2 and 6), ST5A or AT19A was phosphorylated to similar extent as the wild type (Fig. 7a, lanes 8 and 10). These mutant Claspins were also analysed for mobility shift induced by coexpression with Cdc7-ASK (Fig. 7b). Mobility shift, caused by phosphorylation and observed in the wild-type Claspin after overexpression of Cdc7-ASK, was reduced with the ST27A mutant, but was not appreciably affected with ST5A or AT19A (Fig. 7b, lanes 3, 7, 9 and 11). These results show that aa903–1,120 contain major phosphorylation sites, but that other segments are also phosphorylated by Cdc7.

In contrast, Cdc7 did not phosphorylate DE/A mutant (Fig. 7a, lanes 4) or the internal deletion mutants lacking AP (Cdel1 and Cdel7; Fig. 7a, lanes 14 and 20), and no mobility shift was observed on the DE/A mutant after coexpression with Cdc7-ASK (Fig. 7b, lanes 4 and 5). These results indicate that the interaction with Cdc7-ASK through the AP is essential for phosphorylation of Claspin by this kinase, and multiple sites are phosphorylated.

The ST27A and ST27E mutants were expressed in 293T cells and pull-down assays were conducted, as above (Fig. 7c, lanes 9 and 10). They interacted with TopBP1, Cdc45 and Cdc7, suggesting that phosphorylation of this segment per se does not affect the interaction of Claspin with replication factors. ST27E interacted with Cdc7 more strongly than the wild-type Claspin did, suggesting that Cdc7–Claspin interaction may be augmented by Cdc7-mediated phosphorylation of the Claspin.

We then examined the effect of Cdc7-mediated phosphorylation on the interaction between the N-terminal and C-terminal segments of Cdc7. The C-terminal polypeptides (#13, aa897–1,209) and the N-terminal polypeptide (#25, aa1–896) were coexpressed in 293T cells with or without Cdc7-ASK coexpression, and pulled down with Flag-tag attached to #25. The level of the coimmunoprecipitated #13 polypeptide was significantly reduced in the presence of the wild-type Cdc7-ASK (Fig. 7d, compare lanes 12 and 13), but was not affected by a kinase-dead Cdc7-ASK (Fig. 7d, lane 14). These results indicate that Cdc7-ASK is recruited to #13(C)–#25(N) complex, phosphorylates N-terminal segments, resulting in the inhibition of the N–C interaction, that may lead to increased DNA and PCNA bindings.

## Discussion

Claspin is a conserved factor that functions in replication checkpoint as an adaptor protein. It also functions at a DNA replication fork to facilitate the replication fork movement. Indeed, it was identified as a component for ‘Replisome Progression Complex' along with other replication fork factors^32^. However, the precise roles of Claspin at the replication fork are still unclear, let alone its potential roles in initiation. Here, we have generated *Claspin* conditional knockout cells and mice, and used them to evaluate the functions of Claspin under normal course of DNA replication. We have identified AP, a segment rich in acidic residues near the C terminus of Claspin, that is crucial for Claspin functions for unperturbed growth of normal cells. We have shown that this segment serves for recruitment of Cdc7 which plays dual roles; phosphorylation of substrates crucial for initiation and regulation of Claspin functions through modulating the intramolecular interaction between the C-terminal and N-terminal segments. Previously, a docking sequence for Cdc7 kinase was identified on budding yeast Mcm4, (DDD; aa175–333) which is a crucial substrate of Cdc7 kinase *in vivo*^29^. The human Mcm4 sequence corresponding to DDD has ∼30% identity, but it is not known whether this sequence interacts with Cdc7. It was also recently reported in budding yeast that Tof1 and Csm3 (Tim-Tipin in higher eukaryotes) recruit Cdc7 to the replisome during the course of premieotic DNA replication, enabling it to phosphorylate a key substrate for DSB formation^33^. Thus, Cdc7 may be recruited to its substrates through varied ‘recruiter' molecules for efficient and timely phosphorylation events. During the initiation of DNA replication in higher eukaryotes, Claspin may assume this role. Recruitment of Cdc7 by Claspin may add another layer of complexity to regulation of origin firing.

Cdc7 is a conserved kinase and it is well established that Cdc7-mediated phosphorylation of Mcm is a crucial event for initiation of DNA replication^22^,^28^,^29^,^34^,^35^,^36^,^37^. Cdc7-mediated phosphorylation triggers the assembly of initiation complex; namely ‘fires' the replication origins. Thus, selective recruitment of Cdc7 kinase to preformed pre-RCs could be a major determinant for replication timing regulation. Here, we show that AP is required and sufficient for binding to Cdc7 kinase (Fig. 2). It was previously reported that Cdc7-Drf1 (a second subunit of ASK; ASKL1) binds to Claspin in *Xenopus* egg extracts^38^. This binding was reported to be mediated by a segment encompassing one of the Chk1 binding boxes previously identified^14^. AP that was identified in this work does not overlap with this sequence, although the critical segment that may be phosphorylated by Cdc7 overlaps with it (aa903–1,120; Fig. 7a).

AP is rich in acidic residues to which Cdc7 kinase generally has affinity. We further showed that the DE/A mutant of Claspin which cannot bind to Cdc7 is defective in phosphorylation of not only Claspin itself but also crucial substrates of Cdc7, Mcm subunits (Figs 3f and 7a,b). Requirement of Claspin for efficient phosphorylation of Mcms was observed not only in mouse embryonic fibroblast cells but also in Claspin-depleted normal human fibroblast cells (Supplementary Fig. 2). In contrast, loss of Cdc7-mediated Mcm phosphorylation was not observed in Claspin-depleted cancer cells, including HeLa and U2OS cells, consistent with previous reports (Supplementary Fig. 2)^25^. This could be related to the overproduction of Cdc7-ASK in cancer cells^18^,^39^,^40^,^41^(Supplementary Fig. 7), which may overcome the requirement of Claspin for recruitment of Cdc7 kinase to critical substrates. Alternatively, there may be intrinsic differences in assembly and activation of pre-RCs between normal and cancer cells. It is interesting to speculate that Claspin serves as a safeguard for regulated initiation of DNA replication in normal cells. In cancer cells, this protective measure is somehow overrided, which would contribute to their unregulated growth.

It was reported in *Xenopus* egg extracts that chromatin binding of Claspin depends on loading of Cdc45 (ref. 6). In *Saccharomyces cerevisiae*, chromatin binding of Mrc1 was reported to be dependent on Dbf4 (ref. 42). In contrast, our results in normal cells show that Claspin is needed to recruit Cdc7 kinase for pre-RC phosphorylation. Claspin may be present at origins before helicase activation so that Cdc7 could be recruited and the pre-RCs are converted to initiation complex and eventually to active replication forks. Consistent with this prediction, chromatin binding of Claspin was not affected by depletion of Cdc7 in normal human dermal fibroblast cells (Supplementary Fig. 8). It was also reported before in fission yeast that Mrc1 may mark the early firing origins by binding to them in the pre-initiation stage^43^.

The DE/A mutant is partially defective also in interaction with Cdc45, TopBP1 and other replication factors (Figs 3b and 7c). This could be secondary effect of loss of Cdc7-mediated critical phosphorylation of the Mcm complex, which would be required for subsequent recruitment of Cdc45 and other replisome factors to pre-RC. However, Claspin interacted with TopBP1, Pol δ and Cdc45 even in Cdc7-depleted cells (Supplementary Fig. 9). Furthermore, purified Claspin could interact with purified Pol ɛ and Cdc45 in coimmunoprecipitation assays (Supplementary Fig. 10; also reported in ref. 13). Interestingly, both Pol ɛ and Cdc45 can interact with DE/A mutant as well *in vitro*. These results suggest that Claspin can interact with these replication factors in a manner independent of Cdc7 function. Among the replisome components, PCNA and Tim/Tipin are distinct in that they interact with the N-terminal segment of Claspin, and their interactions are not significantly affected by the DE/A mutation (Fig. 3b). These interactions may not only be important for assembly of efficient replication fork machinery and swift response to fork stress but also for enrichment of many replication factors on the chromatin, which may facilitate the assembly of the initiation complex after the firing by Cdc7 kinase.

The second role of AP is mediated by its interaction with the N-terminal segment of Claspin. We have found that DNA-binding activity of Claspin is significantly increased upon truncation of the C-terminal segment (Fig. 4). Further analyses showed that a mutant Claspin carrying the internal deletion of the AP segment bound to DNA with high affinity. Similarly, the DE/A mutant also bound to DNA with much higher affinity and with higher salt resistance than the wild-type Claspin did (Fig. 4c,d). The DE/A mutation in the C-terminal polypeptide resulted in almost complete loss of interaction with the N-terminal polypeptide (Fig. 6b). Thus, AP inhibits DNA-binding activity most likely through interacting with the N-terminal domain. Consistent with this prediction, addition of the C-terminal polypeptide, but not the DE/A mutant, led to inhibition of DNA binding by the N-terminal polypeptide (Fig. 6c).

Binding of PCNA to Claspin was also suppressed by the C-terminal polypeptides containing AP (Fig. 6d). Claspin was previously reported to be a monomer^15^, and thus, the N–C interaction may be most likely intramolecular. Thus, AP regulates the DNA- and PCNA-binding activities through intramolecular interaction with an N-terminal segment. This interaction is probably mediated by ionic interaction between the acidic segment and the basic segment found in the N-terminal segment of Claspin (Fig. 6a,c). This speculation is supported by the finding that DE/A mutation in AP led to the loss of the interaction and suppression (Fig. 6b,c).

It has been reported that Claspin is one of the substrates of Cdc7 kinase^18^,^19^. *In vitro* Cdc7 kinase assays with various polypeptides derived from Claspin indicated that AP is required for efficient phosphorylation of Claspin (Fig. 7a). This is due to loss of recruitment of Cdc7 kinase, since DE/A mutation similarly led to complete loss of phosphorylation, whereas ST/A substitutions in selective segments surrounding AP resulted in only partial loss of phosphorylation. *In vivo*, Cdc7-induced mobility shift (caused by phosphorylation) of Claspin is also almost completely lost in the DE/A mutant (Fig. 7b).

Claspin may recruit Cdc7 at the onset of S phase through AP and the recruited Cdc7 phosphorylates Mcm as well as Claspin. This phosphorylation may trigger the dissociation of the N-terminal segment of Claspin from the AP. Indeed, Cdc7-ASK downregulated the N–C interaction *in vivo* (Fig. 7d). Cdc7 phosphorylates not only the C-terminal segment but also the basic N-terminal segment of Claspin^19^ and this phosphorylation may decrease the ionic N–C interaction. Thus, Cdc7-ASK may activate DNA and PCNA bindings of Claspin through AP-mediated phosphorylation.

Although the interaction between Claspin and PCNA has been known^27^, the PIP motif responsible for this interaction has not been known. We have identified a PIP motif that is required for Claspin–PCNA interaction (Fig. 5). The sequence is slightly deviated from the consensus sequence but amino-acid replacements at the conserved residues resulted in complete loss of PCNA binding both *in vivo* and *in vitro*.

Binding of PCNA to Claspin is required for efficient BrdU incorporation and replication checkpoint activation (Fig. 3). In addition to its potential role in local enrichment of PCNA before initiation, Claspin–PCNA interaction may play a role in efficient fork progression. It is of interest to note that Claspin associates with Tim-Tipin through its N-terminal segment. Claspin-Tim was reported to be required for HU/ultraviolet-induced PCNA ubiquitination^44^. Thus, Claspin may be a platform on which PCNA is ubiquitinated. During the normal course of replication, PCNA clamp may provide a link between the replisome and replication stress surveillance machinery. A similar role of PCNA was suggested for APE2, an adaptor molecule which links oxidative stress to Chk1 activation^45^.

In summary, we report here a novel role of Claspin as a recruiter of Cdc7 kinase and a crucial role of its C-terminal acidic patch for the recruitment. AP serves as a binding pad for Cdc7 kinase and also regulates DNA and PCNA bindings through intramolecular interaction with the N-terminal segment. The recruited Cdc7 not only phosphorylates critical substrates for initiation but also phosphorylates Claspin to disrupt the intramolecular interaction which would activate DNA and PCNA bindings. It was recently reported that TopBP1 is regulated by intramolecular interaction, which is disrupted by acetylation, causing its BRCT to be recognized by other phosphorylated proteins (Rad9 or Treslin)^46^. Intramolecular interaction in Rad9 was also reported to regulate its binding to DNA and TopBP1 (ref. 47). Thus, regulation by intramolecular interaction may be a common mechanism to switch on/off functions of proteins.

## Methods

### Cell lines

293T, HeLa, U2OS and NHDF were obtained from ATCC. Mouse Embryonic Fibroblasts (*MEFs*) were established from E12.5 embryos. Cells were cultured at 37 °C in a 5% CO_2_ humidified incubator in Dulbecco's modified Eagle's medium (high glucose) supplemented with 15% fetal bovine serum (HANA-NESCO), 2 mM L-glutamine, 1% sodium pyruvate, 100 U ml^−1^ penicillin and 100 μg ml^−1^ streptomycin. All the cells have been tested for mycoplasma contamination and turned out to be negative.

### Plasmid construction

The Claspin-encoding DNA fragment (*Xho*I/*Xba*I fragment) of CSII-EF MCS-mAG-TEV-6His-Claspin-3Flag, CSII-EF MCS-6His-Claspin-3Flag or CSII-EF MCS-6His-Claspin-HA plasmid DNA^48^ was replaced by DNA fragments encoding portions of Claspin, amplified by PCR, to express truncated forms of Claspin. To express a Claspin mutant with an internal deletion, two PCR-amplified fragments (*XhoI*-*Bam*HI and *Bam*HI*–Xba*I or *Xho*I-*Nhe*I and *Nhe*I*–Xba*I) were inserted at the *Xho*I-*Xba*I site of CSII-EF MCS-6His-Claspin-3Flag to replace the Claspin insert. The *Eco*RI-*Hpa*I fragment of wild-type or mutant Claspin DNA from mAG-TEV-6His-Claspin-3xFlag was inserted at the *Eco*RI/*Sna*BI site of pMX-IP (Addgene) to construct retroviral expression vectors.

### Southern blotting

Genomic DNA of embryonic stem cells was digested by *Eco*RV or *Eco*RI, run on 0.8% agarose gel at 16 V for 20 h and then transferred to nylon membrane (Genesscreen plus, PerkinElmer). DNA was detected by Southern hybridization using the probes indicated in the figures.

### Transfection of expression vector DNA in 293T cells

The solution ‘A' was made by mixing 1.6 μg of expression plasmid DNA and 100 μl of 150 mM NaCl, and the solution ‘B' was made by mixing 7 μl of 1 mg ml^−1^ solution of PEI (polyethylenimine ‘MAX' (MW25,000; Cat.24765; Polyscience, Inc.)) and 100 μl of 150 mM NaCl. Solution B was added to solution A, mixed well and, after 30 min incubation at a room temperature, the solution was added to six-well plate on which 293T cells had been grown in 2 ml of fresh D-MEM^48^.

### Antibodies and proteins

Antibodies used are as follows. Anti-human Claspin was developed in rabbit against a recombinant protein containing aa896–1,014 of human Claspin produced in *Escherichia coli*. Anti-mouse Claspin was developed in rabbit against a polypeptide LKTNGSSPGPKRSIFRYLES (aa1,296–1,315 of mouse Claspin, 1:500). Anti-PCNA (sc-56, 1:200), anti-Pol α (sc-5920, 1:200), anti-LaminB (sc-6216, 1:200), anti-ATR (sc-1887 1:200), anti-Chk1 (sc-8408, 1:200) and anti-MCM2 (sc-9839, 1:200) antibodies were from Santa Cruz Biotechnology; anti-TopBP1 (A300-111A, 1:1,000) and anti-MCM2 S53 (A300-756A, 1:1,000) antibodies were from Bethyl; anti-Cdc7 (K0070-3, 1:1,000) and anti-Flag (M185-3L, 1:1,000) antibodies were from MBL; anti-Chk1 S345 (#2341, 1:1,000), anti-tubulin (T5168, 1:1,000), anti-myc (04362-34, 1:1,000) and anti-HA (16B12, 1:1,000) antibodies were from Cell Signaling, Sigma-Aldrich, Nacalai Tesque and Abcam, respectively. Anti-MCM4 (1:1,000) was developed in rabbit against GST-fused C-terminal polypeptide of mouse MCM4 (683–861) and were affinity purified against the same polypeptide fused to histidine tag^23^. Anti-Cdc45 (1:1,000) was developed against the recombinant GST-Cdc45 protein expressed in *E. coli* and affinity purified using the His-Cdc45 protein expressed in insect cells^23^. Anti-MCM4 S6T7 (1:1,000) antibody was developed in rabbit against the oligopepetide CMSSPASTPSRRGSRRG (1st to 16th amino acid of human or mouse MCM4), in which both 6th serine and 7th threonine were phosphorylated. Antibody was affinity purified using non-phosphorylated oligonucleotides to remove the antibody reacting with the non-phosphorylated polypeptide^23^. Rabbit anti-Tim (1:1,000) was generated by immunizing rabbit with the C-terminal half (603–1,208 amino acids) of the human Tim and were affinity purified using HiTrap *N*-hydroxysuccinimide-activated HP (GE Healthcare)^49^. Anti-Pol δ (8A5-E3 (ref. 50), 1:1,000) and anti-Pol ɛ (ATCC, CRL-2284, 1:1) antibodies were prepared from the hybridoma cell lines. DNA polymerase ɛ was prepared by infecting insect High5 cells with baculoviruses encoding 6His-tagged human Pol ɛ p261 for 48 h at 27 °C (ref. 51), and Cdc45 was prepared by overexpressing 6His-Cdc45-HA in 293T cells.

### Genotyping from mouse tail

About 1 cm mouse tails were incubated in 50 mM NaOH (in 60 μl) and boiled for 1 min. After spin down of insoluble materials, the supernatant was neutralized by addition of 50 μl 1 M Tris–HCl (pH 8.0) and 1 μl of the resulting solution was used for PCR reaction. Mice were housed in the pathogen-free animal facility of the Tokyo Metropolitan Institute of Medical Science in accordance with the animal care standards of the institution.

### Genotyping from MEF

MEFs were lysed in 500 μl lysis buffer (50 mM Tris–HCl (pH 8.0), 100 mM NaCl, and 20 mM EDTA) containing 150 μg ml^−1^ Proteinase K for 20 h at 55 °C, followed by phenol/chloroform (1:1) extraction, and DNA was recovered by ethanol precipitation. The final pellet was dissolved in 100 μl TE buffer, and used for PCR reaction.

### Knockdown of Claspin or Cdc7 by siRNA

siRNA targeting non-coding mRNA segment of *Claspin* (Supplementary Table 1) or siRNA targeting *Cdc7* (ref. 18) was transfected into indicated mammalian cells using lipofectamine 3,000 (Life Technologies) for 48 h.

### Western blotting

Cell extracts were run on 5–20% gradient SDS–polyacrylamide gel electrophoresis (PAGE; ATTO) or 7.5% SDS–PAGE and then transferred to Hybond ECL membranes (GE Healthcare) followed by western blot analysis with the indicated antibodies. Detection was conducted with Lumi-Light PLUS Western Blotting Substrate (Roche) or SuperSignal West Pico Chemiluminescent Substrate (Thermo) and images were obtained with LAS3000 (Fujifilm).

### BrdU incorporation

To the cells in six-well plates, BrdU was added at 20 mM for 20 min. Cells were harvested and were fixed at −20 °C by 75% ethanol. After wash with wash buffer (0.5% bovine serum albumin in phosphate-buffered saline), cells were treated with 2 N HCl for 20 min and then with 0.1 M sodium borate (pH8.5) for 2 min, both at room temperature. Then, the cells were treated with FITC-conjugated anti-BrdU antibody (BD biosciences, 51–23,614 l) for 20 min at room temperature in the dark, and further incubated with propium iodidide (25 μg ml^−1^) and RNaseA (100 μg ml^−1^) for 30 min at room temperature, followed by analyses with FACS (fluorescence-activated cell sorting).

### Cell fractionation and immunoprecipitation *in vivo*

Cells were lysed in CSK buffer (10 mM PIPES-KOH (pH 6.8), 100 mM potassium glutamate, 300 mM sucrose, 1 mM MgCl_2_, 1 mM EGTA, 1 mM DTT, 1 mM Na_3_VO_4_, 50 mM NaF, 0.1 mM ATP, protease inhibitor-PI tablet (Roche) and 0.5 mM PMSF), containing 0.1% TritonX-100 and 10 units per ml Benzonase (Amersham plc.). After rotating for 60 min in cold room, the supernatants were recovered and the pellets were washed two times and recovered as chromatin-enriched fractions. The supernatants were incubated with anti-Flag M2 affinity beads (SIGMA, A2220) for 60 min at 4 °C. The beads were washed with CSK buffer three times and proteins bound to the beads were analysed by western blotting.

### Growth curve

Cells (1 × 10^5^) of f/− MEFs cells or those stably expressing Claspin or its mutant were treated with Ad-Cre or non-treated for 48 h, and were passaged to new six-well plates. The cells were harvested at the times indicated after the passage and cell numbers were counted at the times indicated.

### Protein purification

293T cells (15 cm dish, 3–5 plates) incubated for 40 h after transfection were harvested and lysed as above. For DE/A mutant, CSK buffer was supplemented with 500 mM NaCl. The proteins bound to anti-Flag M2 affinity beads (Sigma-Aldrich) were recovered from the supernatants and washed by Flag wash buffer (50 mM NaPi (pH 7.5), 10% glycerol, 300 mM NaCl, 0.2 mM PMSF and PI tablet), and bound proteins were eluted with Flag elution buffer (50 mM NaPi (pH 7.5), 10% glycerol, 30 mM NaCl, 200 μg ml^−1^ 3xFlag peptide (SIGMA), 0.1 mM PMSF and PI tablet; see Uno *et al*.^48^ for more details).

### Preparation of labelled Y-fork

^32^P-end-labelled sense oligonucleotide (32mer-dT_32_; 240 ng) was mixed with antisense oligonucleotide (dT_32_-32mer; 300 ng) in 20 mM Tris–HCl (pH 7.4) and 10 mM MgCl_2_, and incubated at 96 °C for 3 min and then at 37 °C for 60 min (ref. 52). The generated ^32^P-labelled Y-fork DNA was isolated from PAGE and dissolved in TE buffer.

### Gel shift assay

Labelled Y-fork DNA (20 fmol) was incubated with purified proteins of indicated amounts in gel shift buffer (10 mM Tris–HCl (pH 7.5), 40 μg ml^−1^ BSA, 1 mM DTT, 50 mM EDTA, 20 mM K-glutamate and 8% glycerol), incubated at 30 °C for 1 h and the samples were applied onto 5% 29:1 PAGE (1 × Tris-Borate-EDTA and 5% glycerol). The gel was dried and autoradigraphed.

### Filter-binding assay

Binding reactions contained ^32^P-labelled Y-fork DNA (80 fmol) and purified wild-type or mutant Claspin protein of indicated amount in 1 × binding buffer (10 mM Tris–HCl (pH 7.5), 40 μg ml^−1^ BSA, 1 mM DTT, 0.5 mM EDTA, 20 mM K-glutamate and 8% glycerol) and were incubated for 1 h at 30 °C. HA membranes (MFTM Membrane Filters (0.45 μm HA), Millipore) were activated by soaking in 4 N KOH solution for 10 min followed by wash in water twice for 10 min each. The processed HA membranes and untreated DE81 membranes were washed in 1 × binding buffer for 1 h. On the filtration unit, a HA membrane (trapping proteins) was placed on top of a DE81 membrane (trapping all the ssDNA) and washed with 3 ml of 1 × binding buffer. Next, a binding reaction mixture was passed through and further washed twice with 3 ml of 1 × binding buffer. In some experiments, filters were washed twice with 1 × binding buffer containing different concentration of KCl. After drying, radioactivity trapped on each membrane was counted by liquid scintillation counter. Radioactivity trapped on the HA membrane was divided by the sum of that on the HA and DE81 membranes and the values were presented as binding activity.

### Pull-down assay *in vitro*

For pull-down assays of PCNA and Cdc7-ASK, recombinant wild-type or mutant Claspin protein was incubated with PCNA (BioAcademia) or Cdc7-ASK, as indicated in each experiment, in the reaction buffer (40 mM HEPES-KOH (pH 7.6), 100 mM K-glutamate, 1 mM MgCl_2_, 1 mM EGTA, 0.01% TritonX-100, 0.1 mM ATP and 0.5 mM PMSF) at 4 °C for 1 h. Anti-Flag M2 affinity beads were added and beads were recovered by centrifugation, washed twice with the reaction buffer. Proteins attached to the beads were analysed by SDS–PAGE and detected by western blotting.

### Evaluation of Cdc7-mediated phosphorylation in cells

Wild-type or mutant Claspin was transfected into 293T cells with or without the combination of pME18S-mycASK and pME18S-HACdc7 (wild type or kinase dead^28^) plasmid DNAs. After 40 h, cells were harvested, and extracts were prepared which, incubated with lambda phosphatase or non-treated, were analysed by western blotting with antibodies indicated.

### *In vitro* kinase assays with Cdc7-ASK

Wild-type or mutant Claspin protein (0.3 pmol) was incubated in the presence or absence of 0.03 pmol Cdc7-ASK complex in the kinase reaction buffer (50 mM HEPES-KOH (pH 7.9), 10 mM MgCl_2_, 2 mM DTT, 10 μM ATP and 1 μCi of [γ-^32^P]ATP) for 30 min at 37 °C. One-forth volume of 5 × sample buffer was added, heated at 96 °C for 3 min and analysed by SDS–PAGE, followed by CBB staining and autoradiogram.

### Glycerol gradient sedimentation

The purified wild-type or mutant Claspin protein was loaded on glycerol gradient (15–35%) in 20 mM Tris–HCl (pH 7.5), 0.5 mM EDTA, 1 mM DTT, 150 mM NaCl, 0.1 mM PMSF and 0.01% TritonX-100, which was centrifuged at 104,900*g* for 16 h at 4 °C in the TL100 rotor with Optima MAX-XP Ultracentrifuge (BECKMAN COULTER). High molecular weight native marker proteins (GE Healthcare) were fractionated under the same condition as size markers. The fractions were analysed by SDS–PAGE and proteins were detected by western blotting (Claspin) or CBB staining (markers).

### Data availability

The data that support the findings of this study are available from the corresponding author upon request.

## Additional information

**How to cite this article:** Yang, C.-C. *et al*. Claspin recruits Cdc7 kinase for initiation of DNA replication in human cells. *Nat. Commun.* 7:12135 doi: 10.1038/ncomms12135 (2016).

## Supplementary Material

Supplementary Information Supplementary Figures 1-27, Supplementary Table 1 and Supplementary Reference

## Figures and Tables

**Table 1:** Crossing of *Claspin* ^w/−^ × *Claspin* ^w/−^.

Embryo	w/w	w/−	−/−	Ratio of −/−
9.5 days	3	3	1	1/7
12.5 days	5	12	0	0/17

Although *Claspin*^−/−^ embryos could be detected at E9.5, no −/− embryos were obtained at E12.5, indicating that *Claspin*^−/−^ embryos die by E12.5.
