# Supplementary material for: Claspin recruits Cdc7 kinase for initiation of DNA replication in human cells
Source: Nat Commun. 2016 Jul 12;7:12135. doi: 10.1038/ncomms12135 (PMC4945878; doi:10.1038/ncomms12135)
Supplement: Supplementary Information — Supplementary Figures 1-27, Supplementary Table 1 and Supplementary Reference [file ncomms12135-s1.pdf]

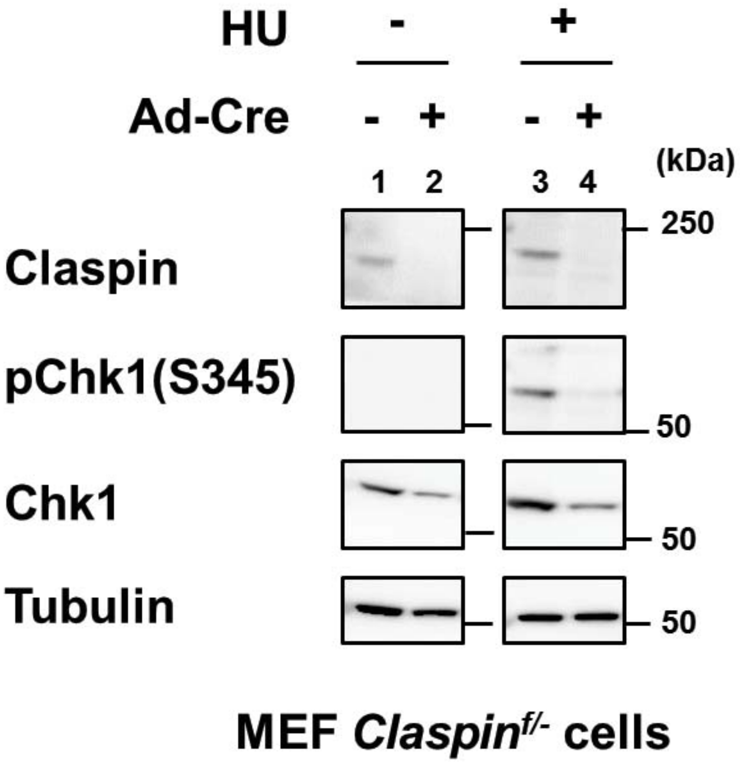

Supplementary Figure 1 (related to Figure 1). Chk1 activation in response to HU is compromised in *Claspin*$^{-/-}$ MEF cells.

Proteins indicated were examined by western blotting in the whole cell extracts of the *Claspin*$^{f/-}$ MEF cells. 2mM HU was added during the last 3 h before the harvest (right) or non-treated (left). Cells were treated with Ad-Cre (+) or untreated (-).

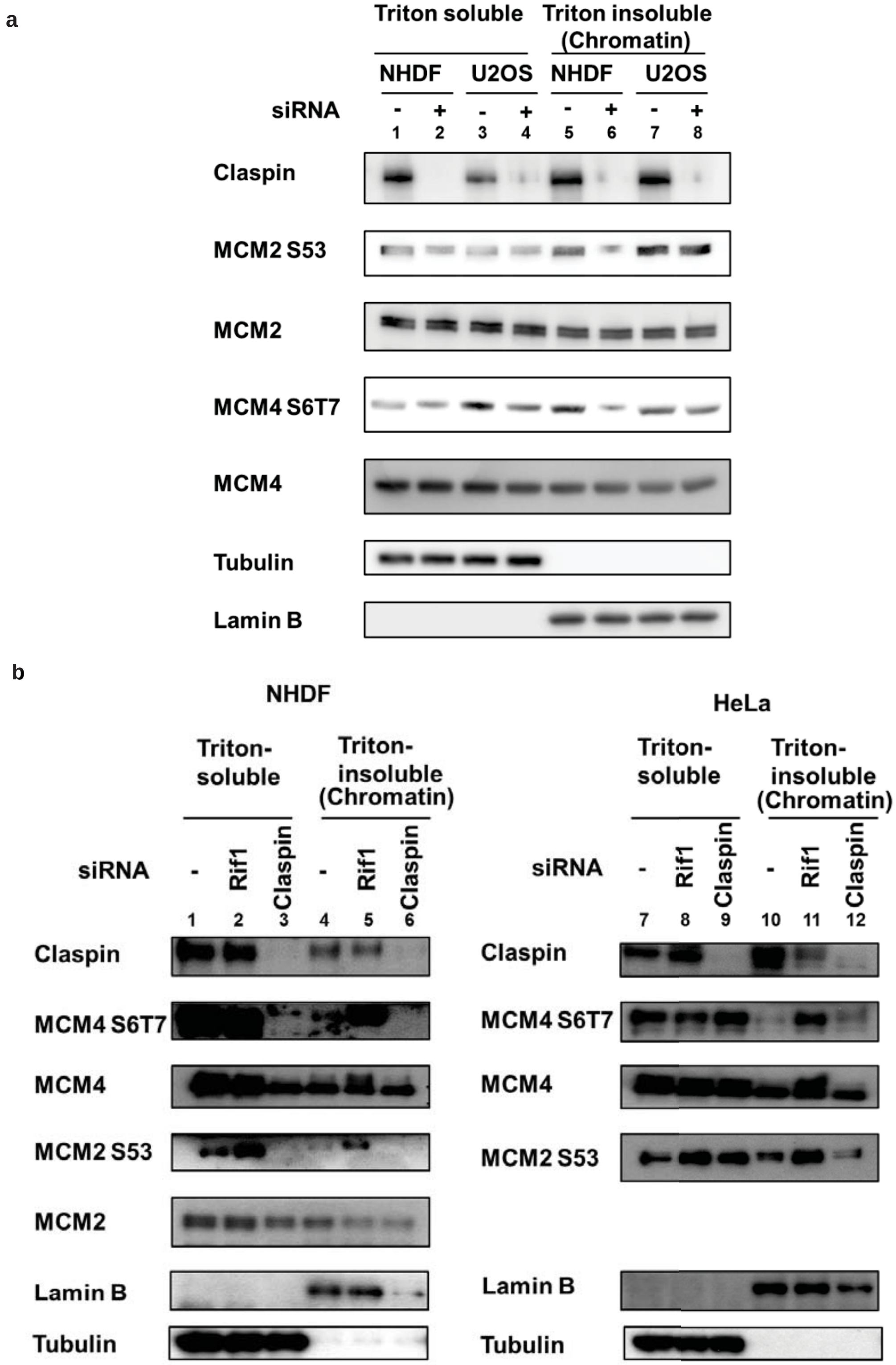

Supplementary Figure 2 (related to Figure 3). Claspin is required for Cdc7-mediated phosphorylation of Mcm subunits in NHDF but not in cancer cell lines.

(**a**) NHDF and U2OS cells were mock-transfected or transfected with *Claspin* siRNA and Triton-soluble and -insoluble fractions were analyzed by western blotting to detect the proteins indicated. (**b**) NHDF and HeLa cells were mock-transfected or transfected with *Rif1* or *Claspin* siRNA and Triton-soluble and -insoluble fractions were analyzed by western blotting to detect the proteins indicated. It has been known that Rif1 depletion increases the Cdc7-mediated Mcm phosphorylation[1].

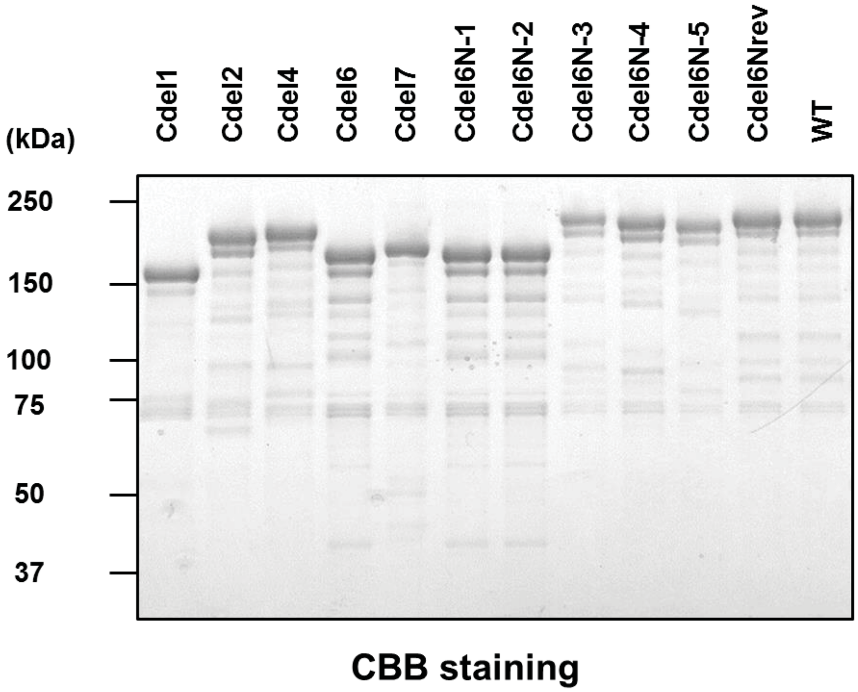

Supplementary Figure 3 (related to Figure 4). Purification of Claspin derivatives carrying internal deletions in the C-terminal region.

Each protein was expressed in 293T cells and purified as described in Methods. 2.5 μg of the purified protein fractions were run on 5-20% gradient gel, and stained with CBB.

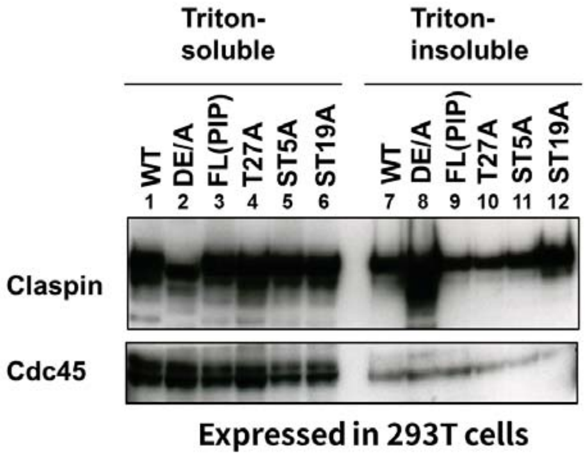

Supplementary Figure 4 (related to Figure 4) Chromatin binding of various mutant Claspin proteins.
293T cells were transfected with plasmid DNAs expressing the wild-type, DE/A, PIP, ST27A, ST5A or ST19A Claspin. Triton-soluble and -insoluble fractions were prepared and were analyzed by western blotting to detect the transfected protein (anti-Flag antibody) and Cdc45.

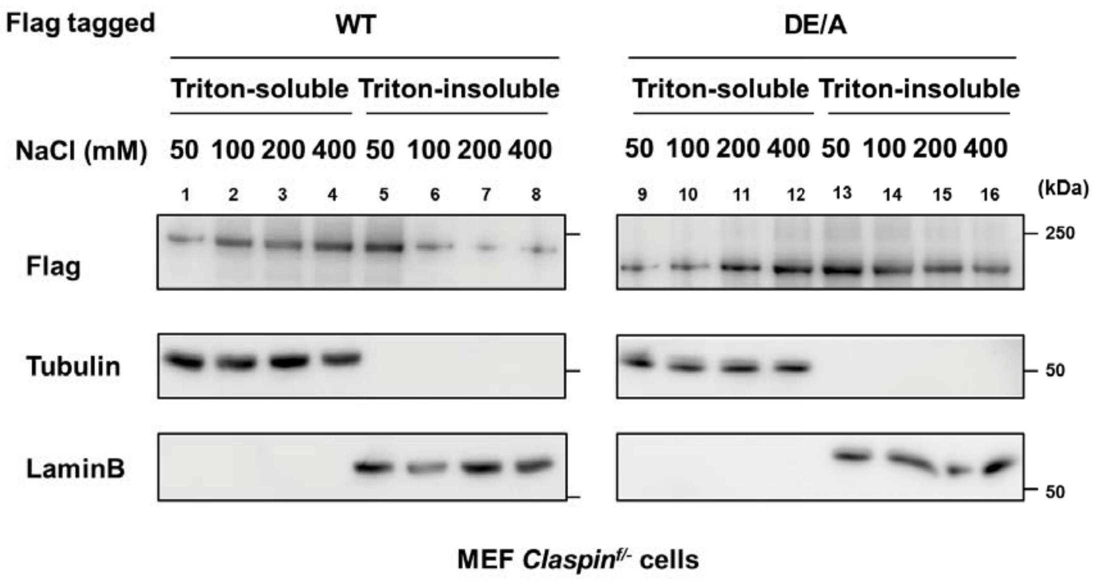

Supplementary Figure 5 (related to Figure 4). Effect of salt concentration on chromatin binding of wild-type and DE/A mutant Claspin.

Claspin$^{f/-}$ MEF cells stably expressing the Flag-tagged wild-type or DE/A mutant Claspin were fractionated into Triton-soluble and -insoluble fractions at different concentrations of NaCl. Each fraction was analyzed by western blotting to detect the proteins indicated.

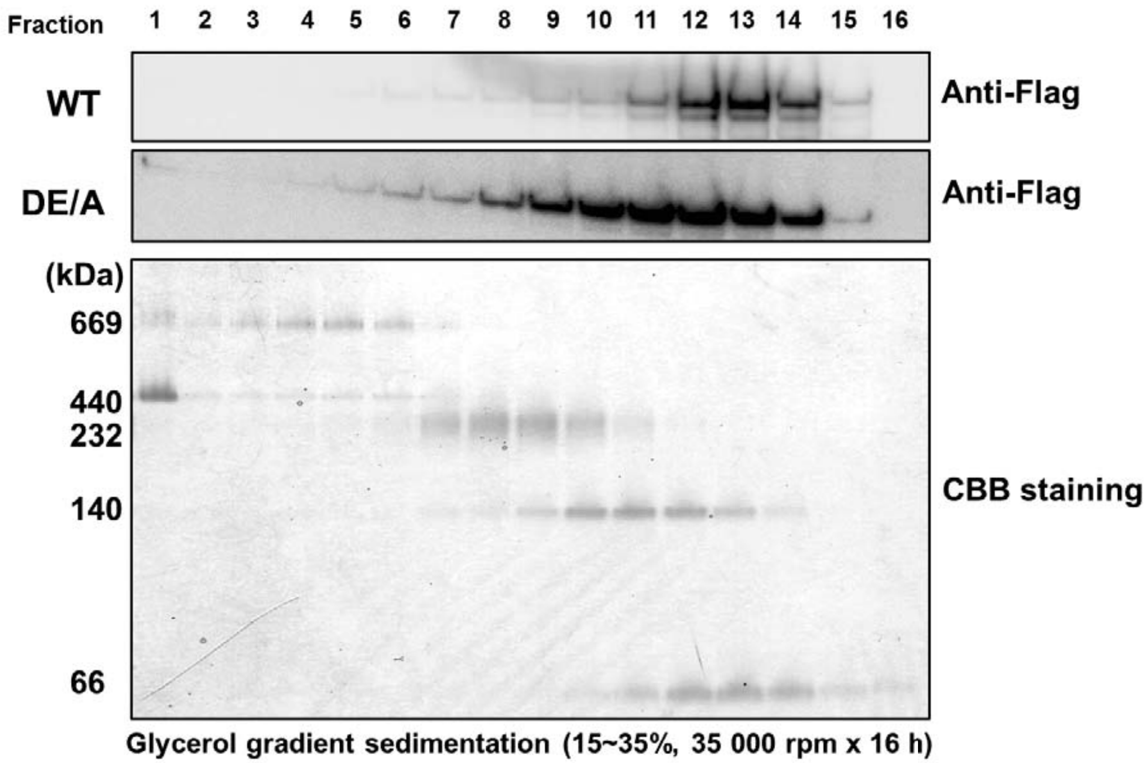

Supplementary Figure 6 (related to Figures 3 and 4) Glycerol gradient analyses of purified wild-type and DE/A mutant Claspin proteins.

Six μg of purified Claspin proteins (wild-type and DE/A) were fractionated by gel filtration column (Superose-6) in SMART system. The buffer used was 20 mM Tris–HCl (pH 7.5), 0.5 mM EDTA, 1 mM DTT, 150 mM NaCl, 0.1 mM PMSF and 0.01% TritonX-100. High molecular weight native marker proteins (GE Healthcare, Inc.) were also fractionated under the same condition. The fractions were analyzed by SDS–PAGE and western blotting with anti-Flag antibody or Coomassie Brilliant Blue (CBB) staining.

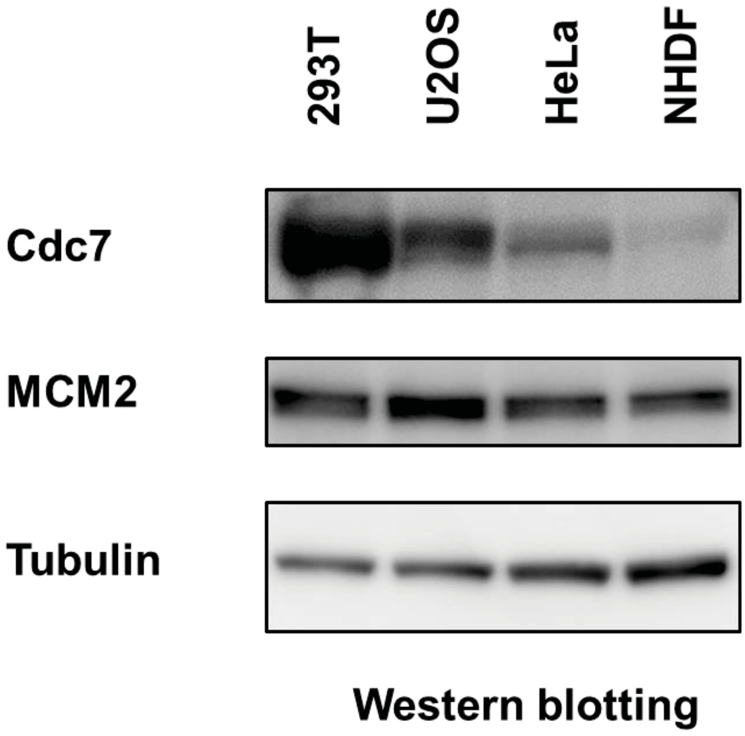

Supplementary Figure 7 (related to Figure 3). Cdc7 is overexpressed in cancer cell lines.
Whole cell extract of 293T, U2OS, HeLa and NHDF cells were analyzed by western blotting to detect the proteins indicated.

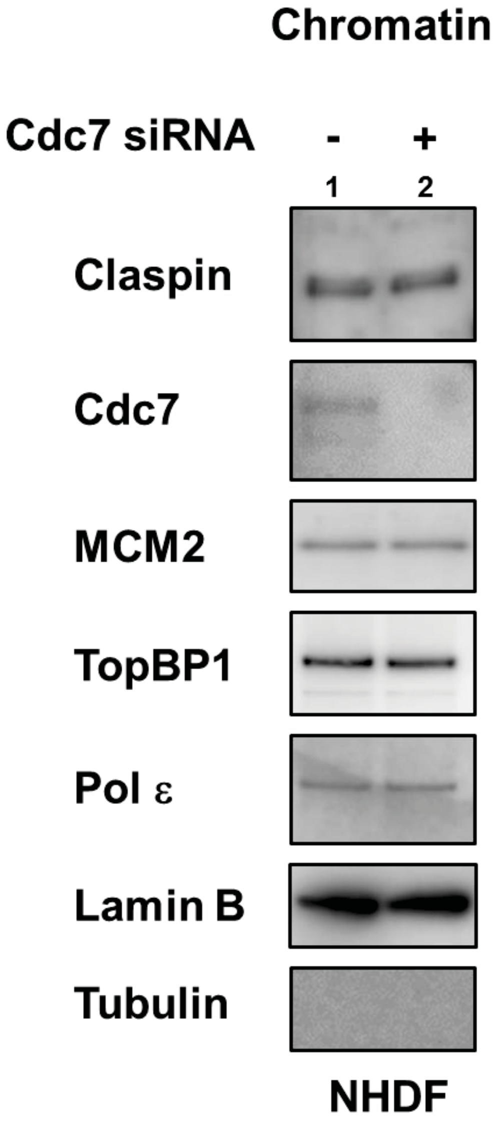

Supplementary Figure 8 (related to Figure 3). Cdc7 depletion does not affect the Claspin binding to chromatin.

Cdc7 was knocked down by siRNA in NHDF cells for 48 h, and cells were fractionated into Triton-soluble and -insoluble fractions. Triton-insoluble fractions were analyzed by western blotting to detect the proteins indicated.

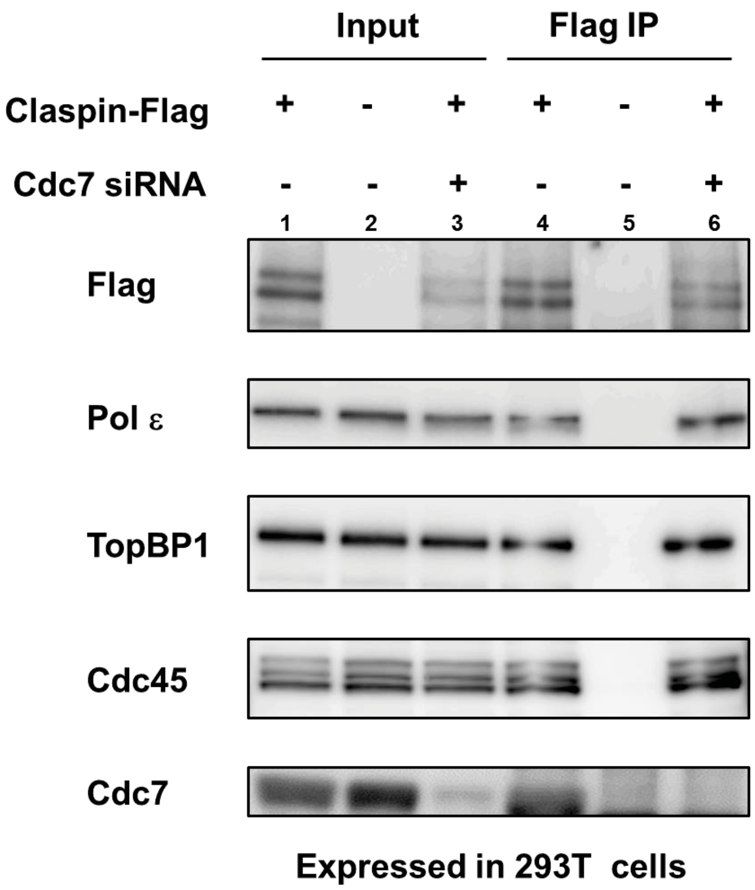

Supplementary Figure 9 (related to Figures 2 and 3). The binding of Pol ε, TopBP1, and Cdc45 to Claspin is independent of Cdc7.

Cdc7 was knockdown by siRNA in 293T cells, and then Flag-tagged Claspin was expressed by transient transfection. The immunoprecipitates with M2 Flag beads were analyzed by western blotting to detect the proteins indicated.

a

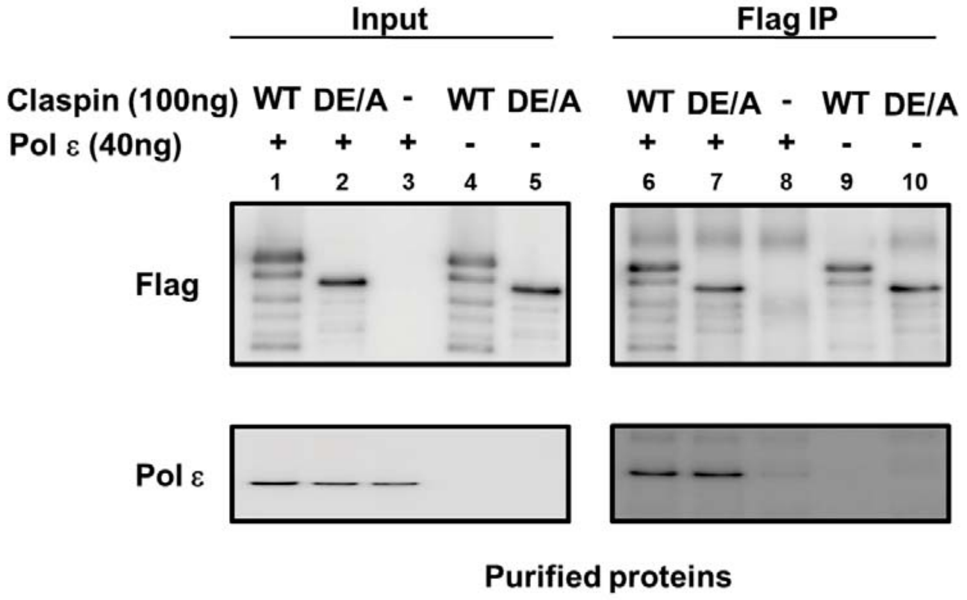

b

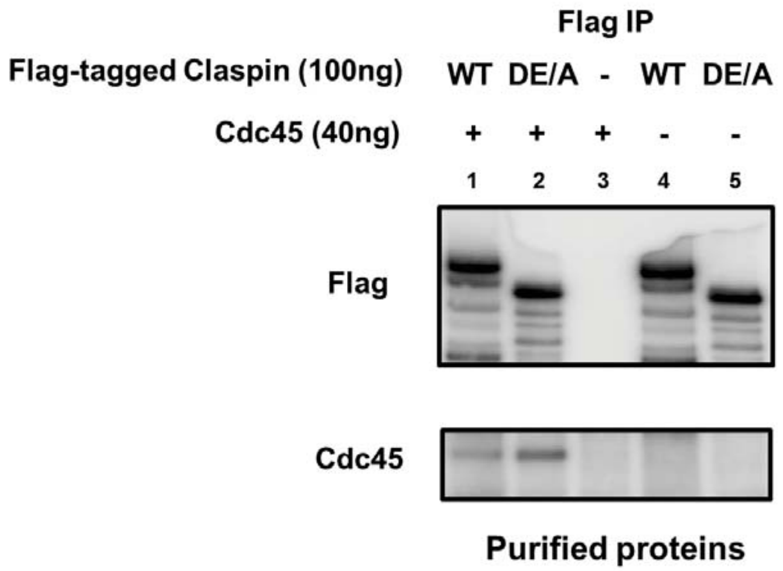

Supplementary Figure 10 (related to Figures 2 and 3). Pol ε and Cdc45 interact with Claspin in a manner independent on AP *in vitro*.

Purified wild-type or DE/A mutant Claspin was mixed with purified Pol ε (**a**) or with Cdc45 (**b**) and pulled down by Dynabeads conjugated with anti-Flag antibody. Immunoprecipitated materials were analyzed by western blotting to detect the proteins indicated.

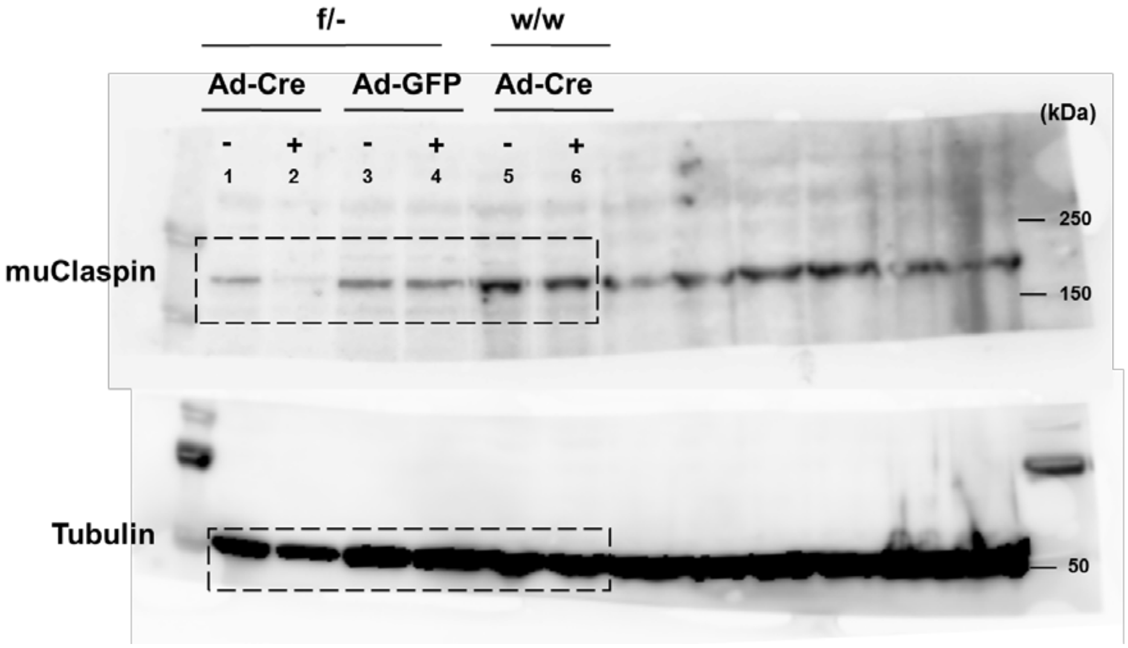

Supplementary Figure 11. Uncropped scans of blots.
Uncropped scans of Figure 1c.

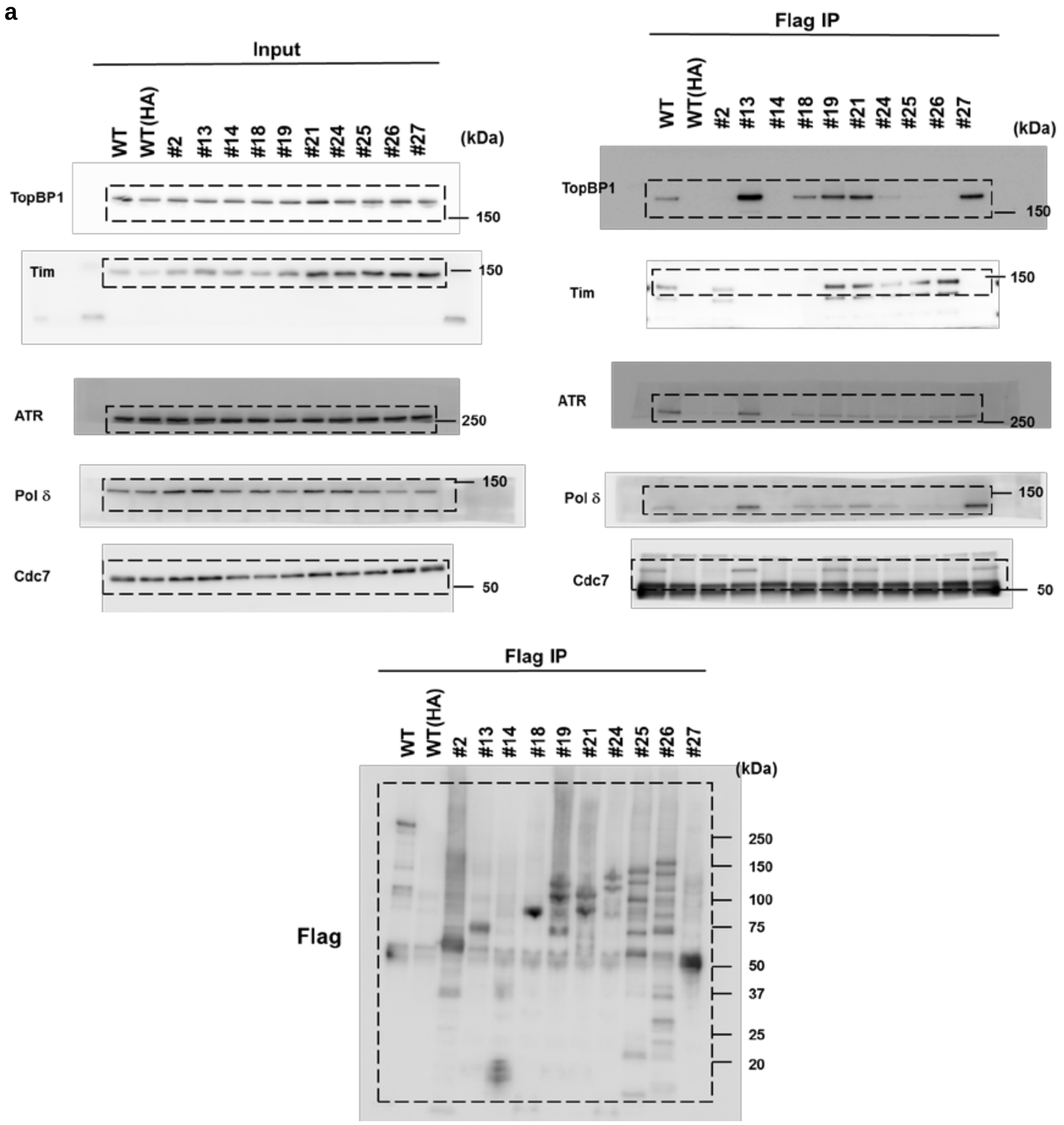

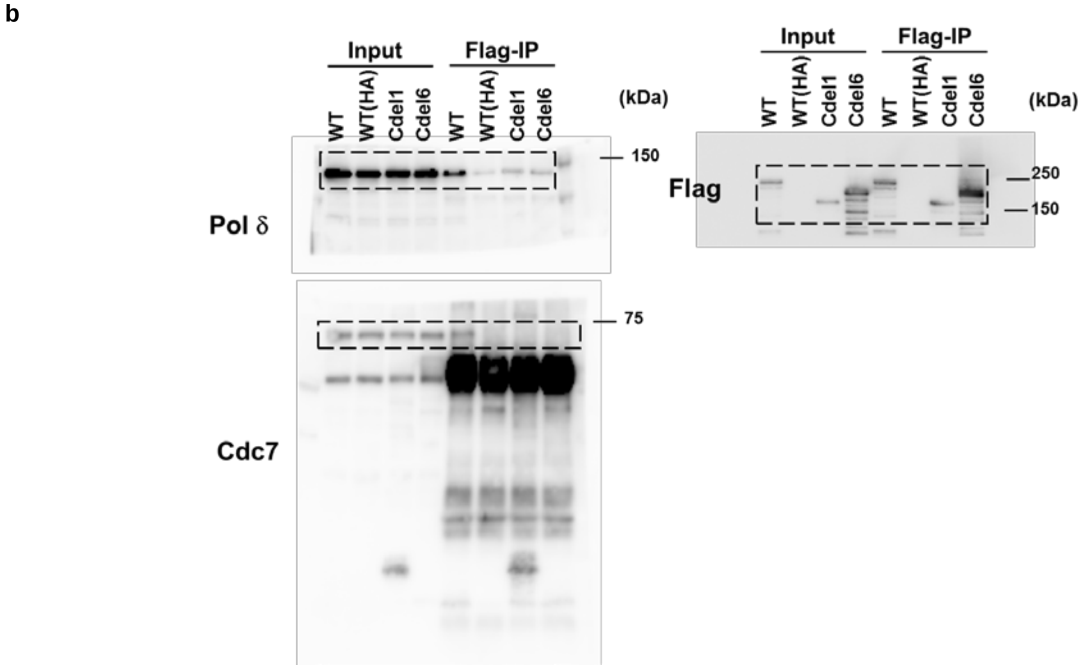

Supplementary Figure 12. Uncropped scans of the most important blots.
(a) Uncropped scans of Figure 2b.(b) Uncropped scans of Figure 2c.

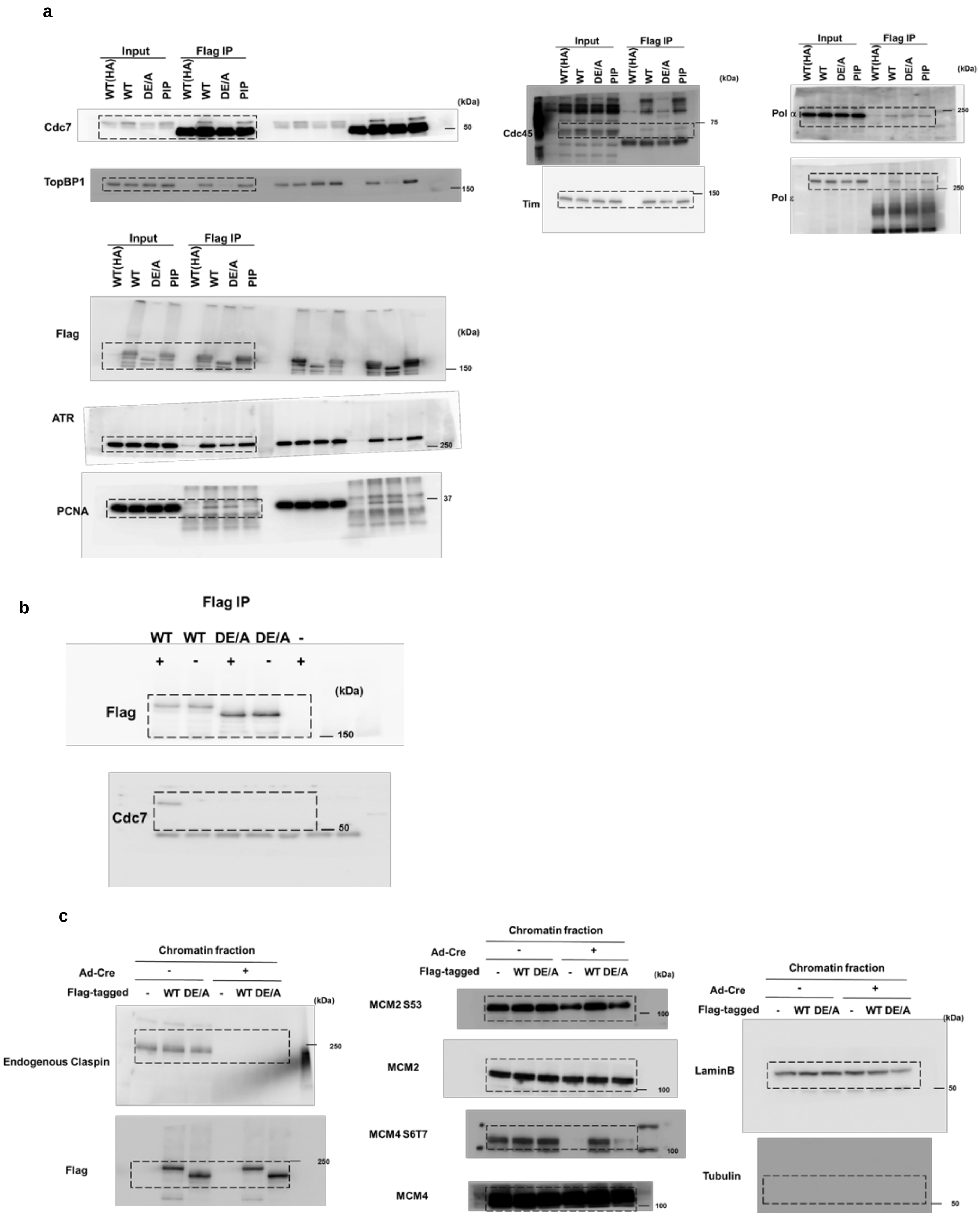

Supplementary Figure 13. Uncropped scans of the blots.

(a)Uncropped scans of Figure 3b.(b) Uncropped scans of Figure 3c.(c) Uncropped scans of Figure 3f.

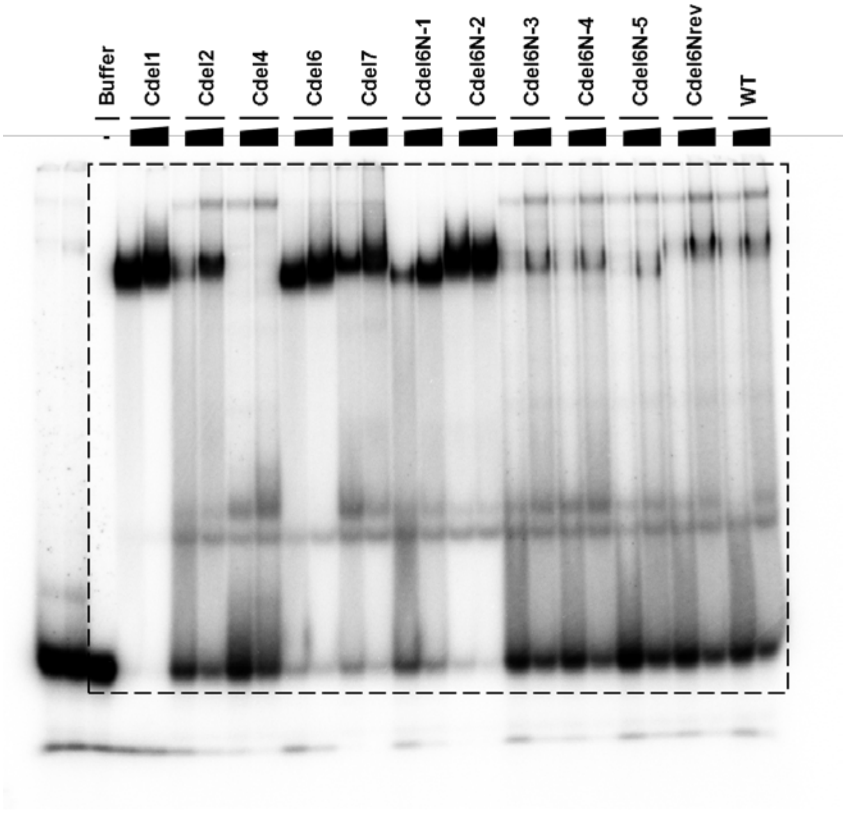

Supplementary Figure 14. Uncropped scans of the blots.
Uncropped scans of Figure 4b.

a

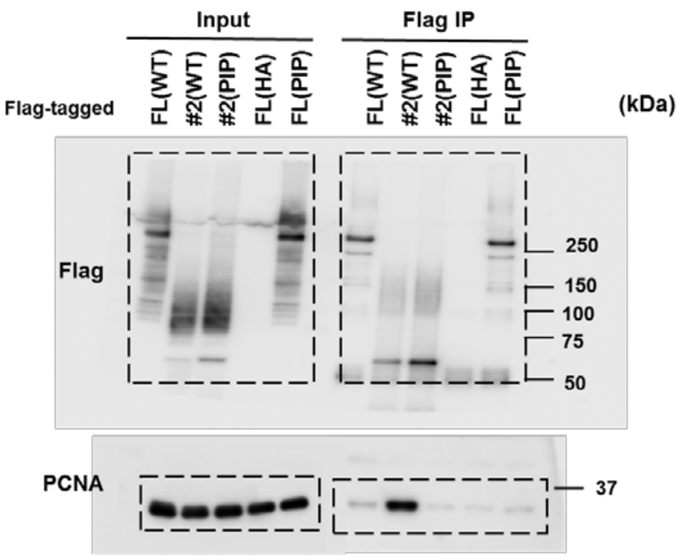

b

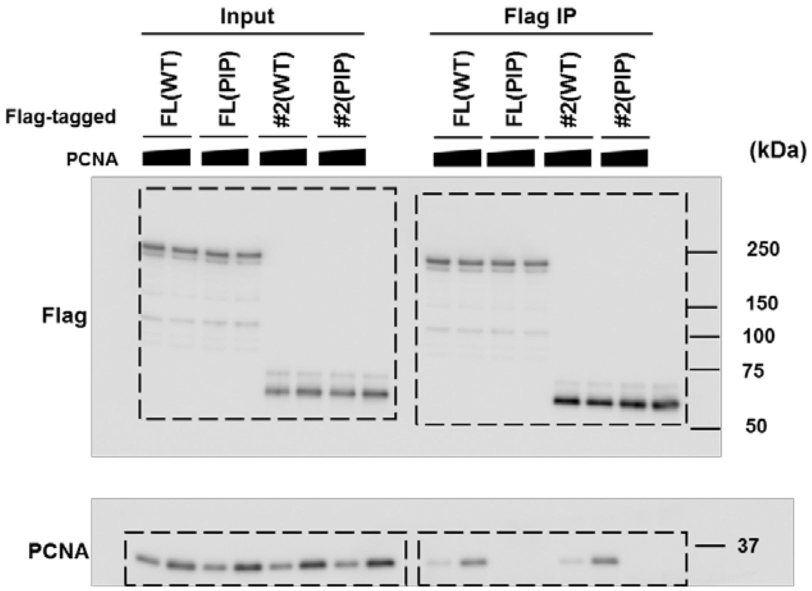

Supplementary Figure 15. Uncropped scans of the blots.
(a)Uncropped scans of Figure 5b.(b) Uncropped scans of Figure 5c.

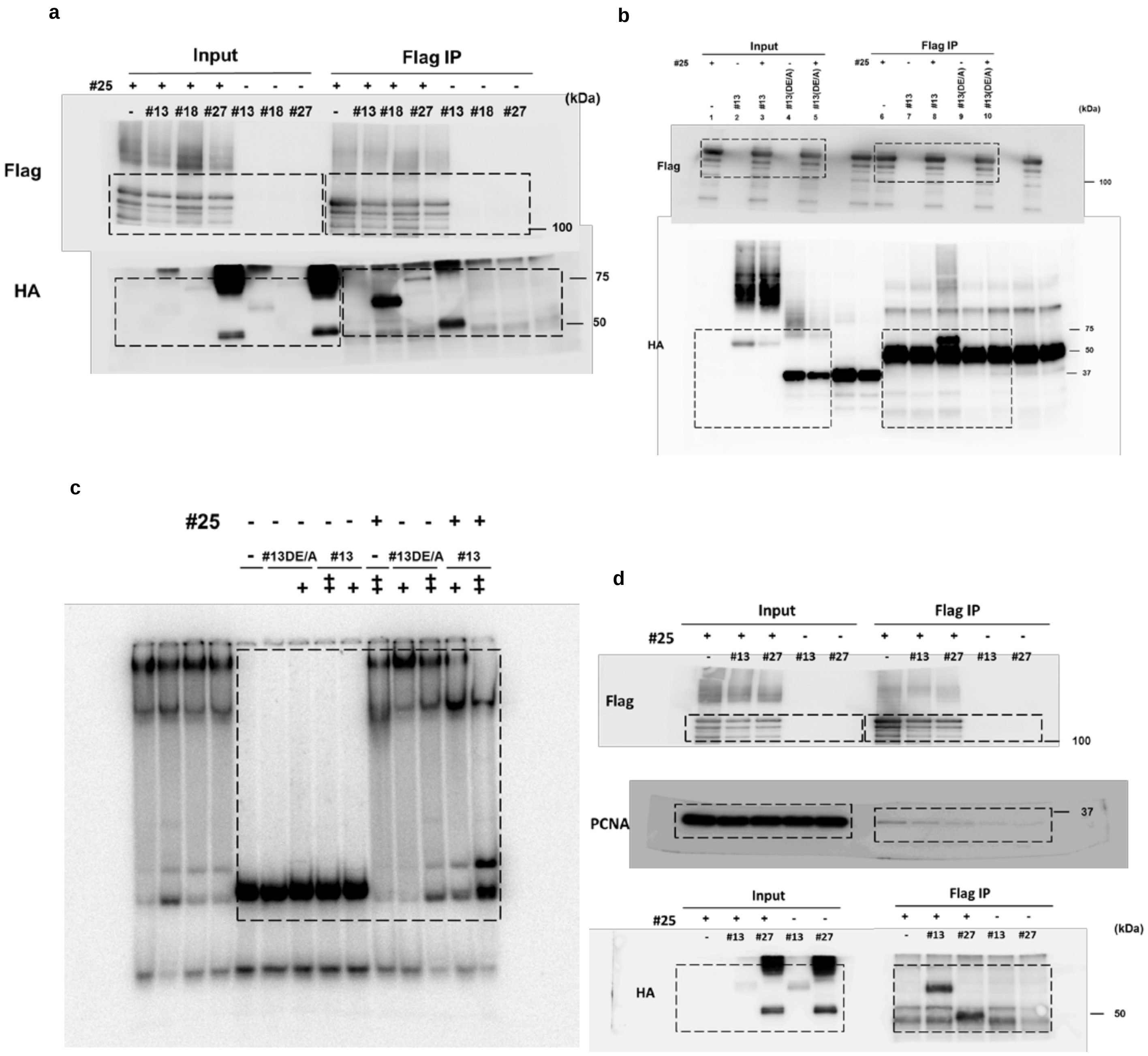

Supplementary Figure 16. Uncropped scans of the most important blots.
(a)Uncropped scans of Figure 6a.(b) Uncropped scans of Figure 6b. (c)Uncropped scans of Figure 6c. (d)Uncropped scans of Figure 6d.

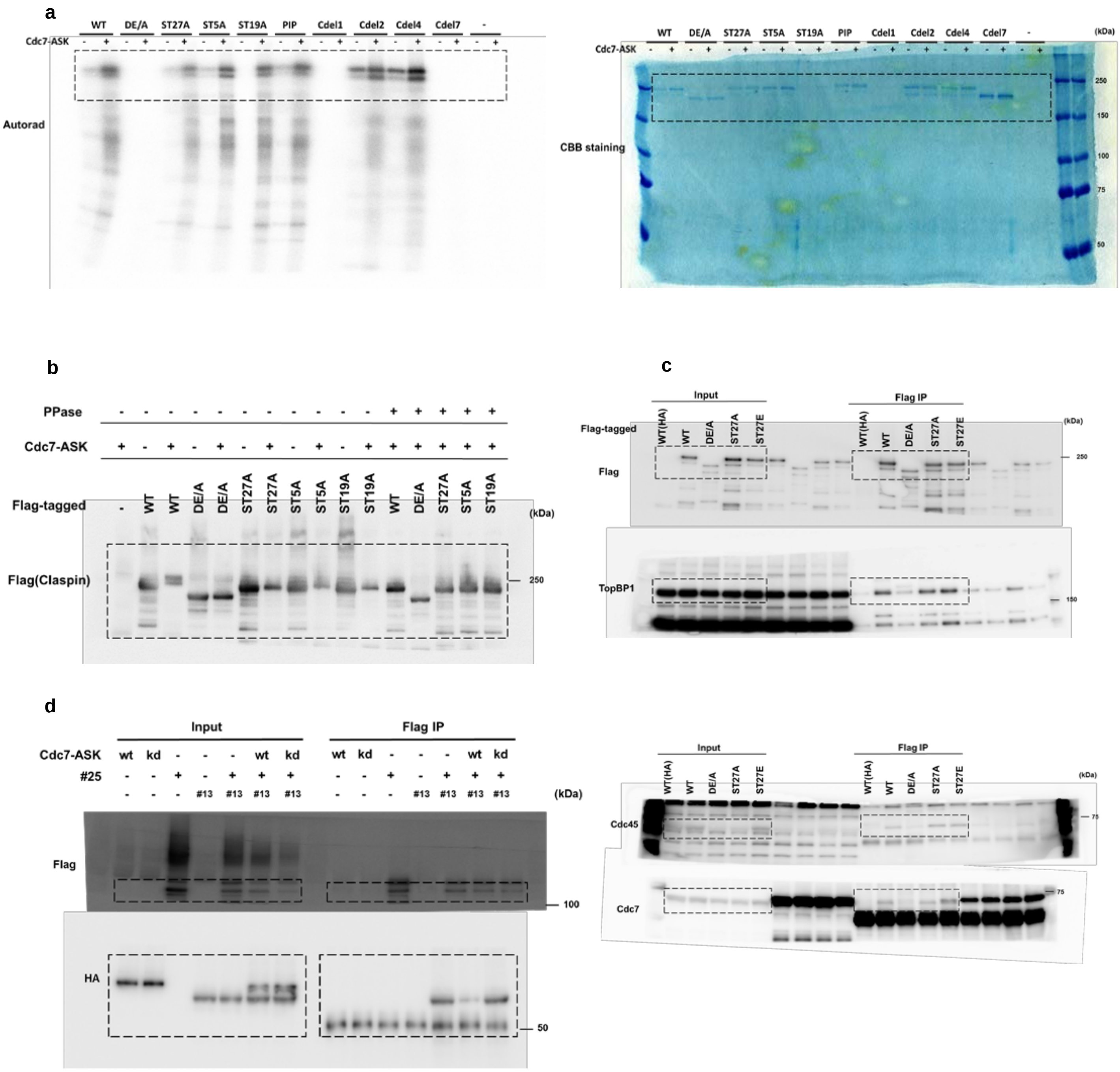

Supplementary Figure 17. Uncropped scans of the blots.

(a)Uncropped scans of Figure 7a.(b) Uncropped scans of Figure 7b. (c)Uncropped scans of Figure 7c. (d)Uncropped scans of Figure 7d.

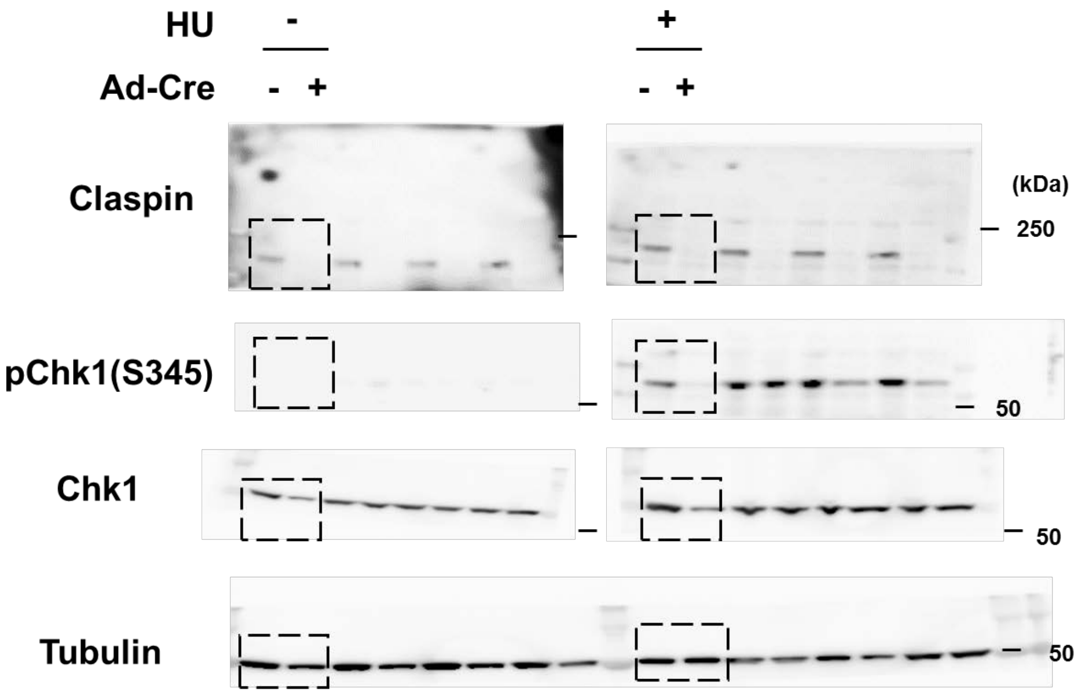

Supplementary Figure 18. Uncropped scans of the blots.
Uncropped scans of Supplementary Figure 1

a

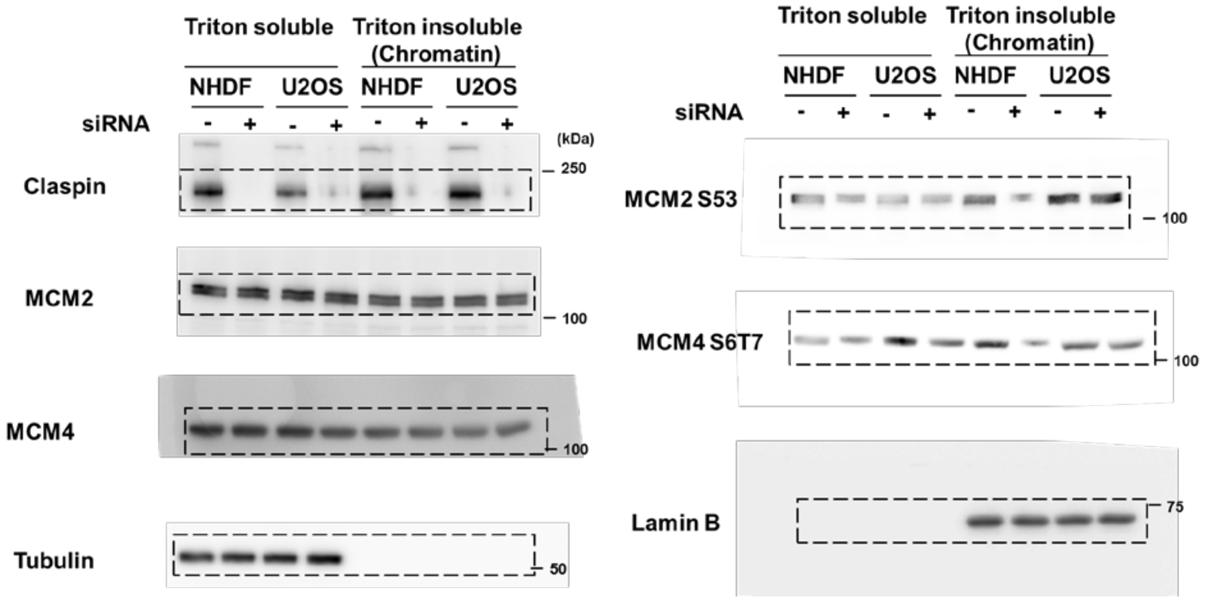

b

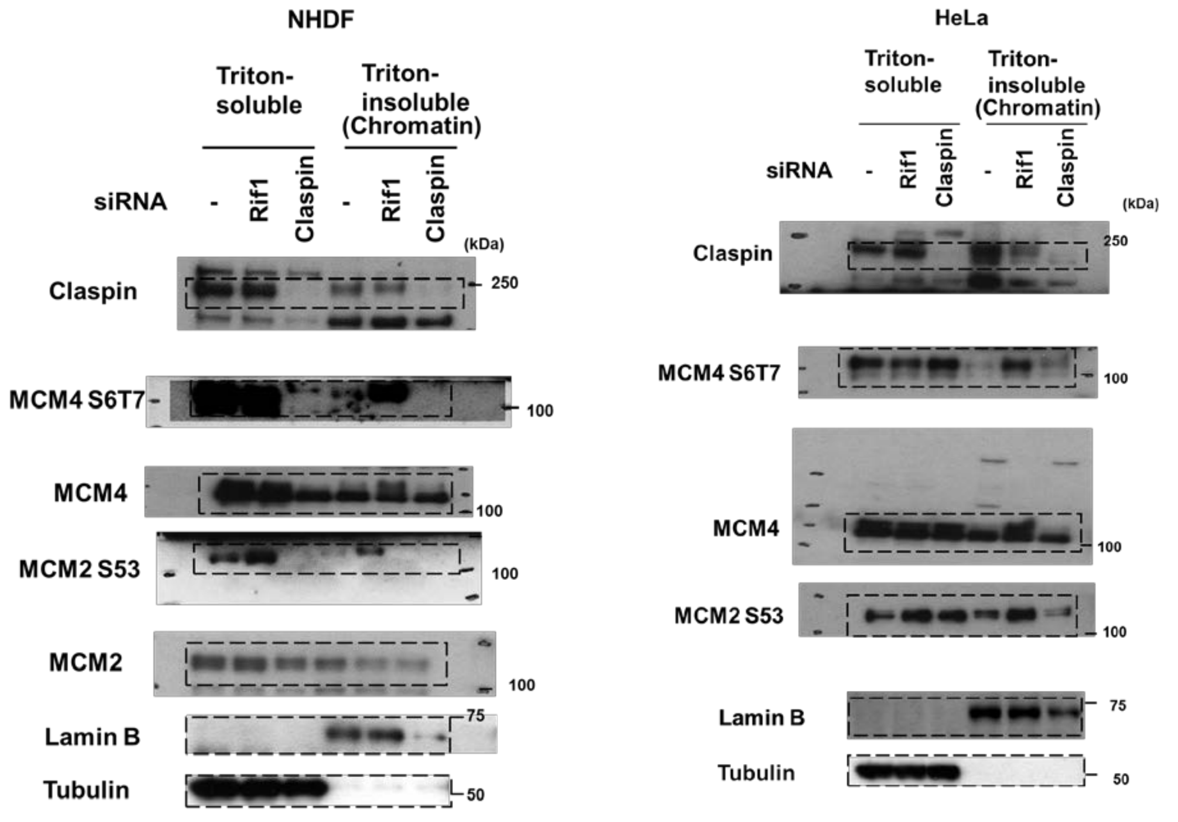

Supplementary Figure 19. Uncropped scans of the blots.
(a) Uncropped scans of Supplementary Figure 2a. (b) Uncropped scans of Supplementary Figure 2b.

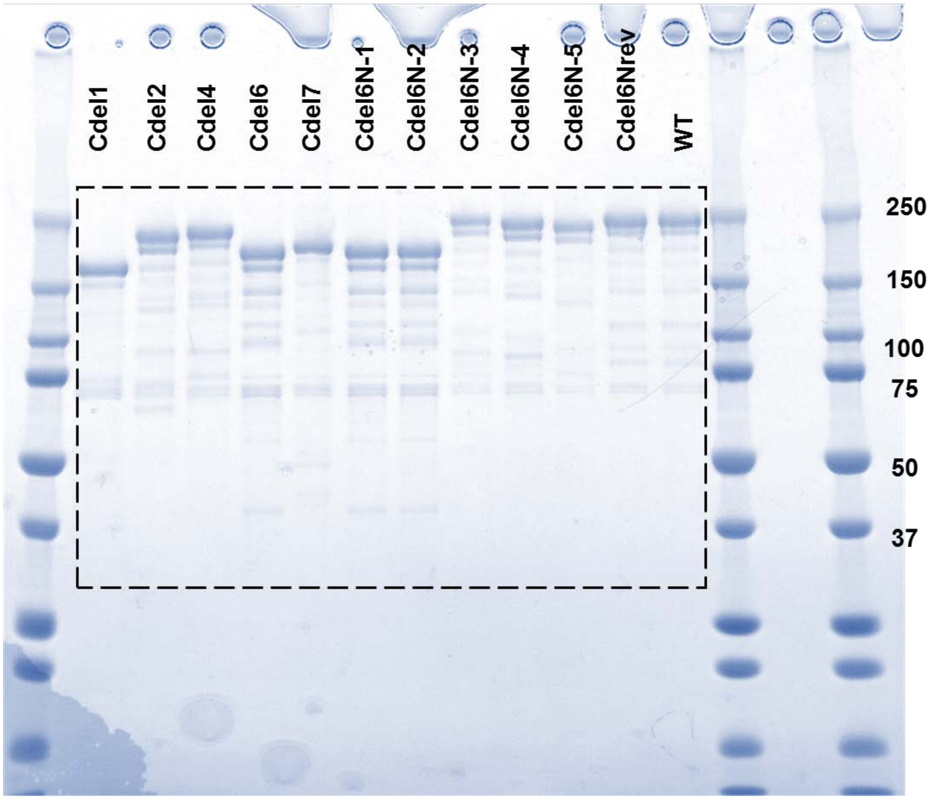

Supplementary Figure 20. Uncropped scans of the blots.
Uncropped scans of Supplementary Figure 3.

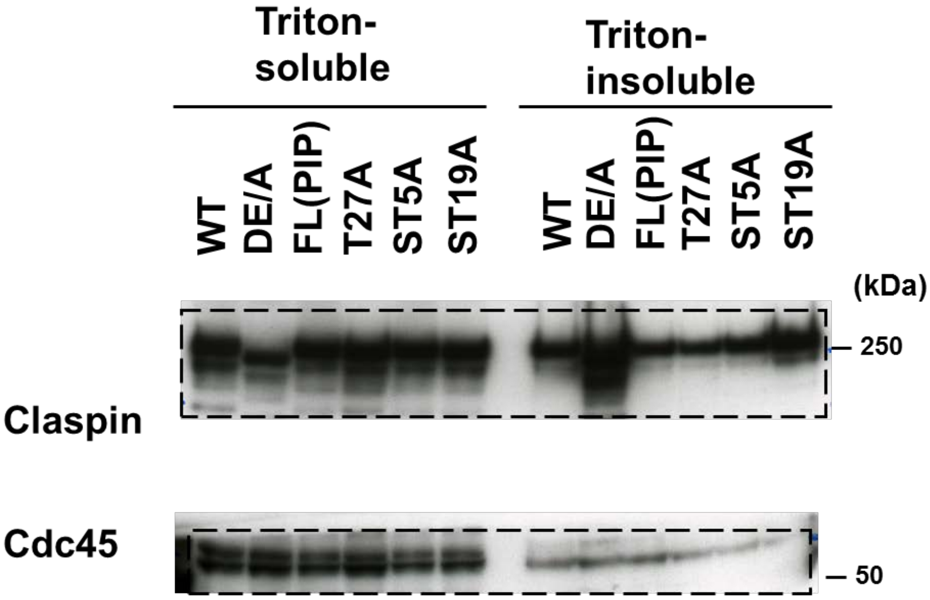

Supplementary Figure 21. Uncropped scans of the blots. Uncropped scans of Supplementary Figure 4.

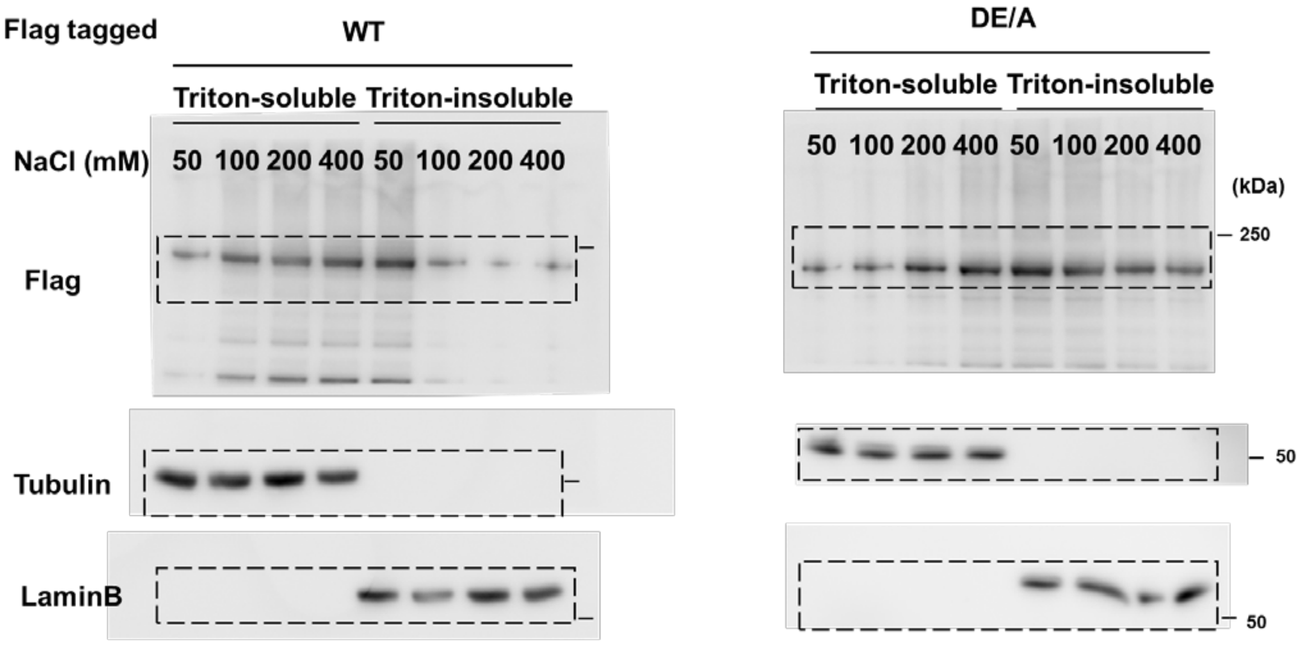

Supplementary Figure 22. Uncropped scans of the blots.
Uncropped scans of Supplementary Figure 5.

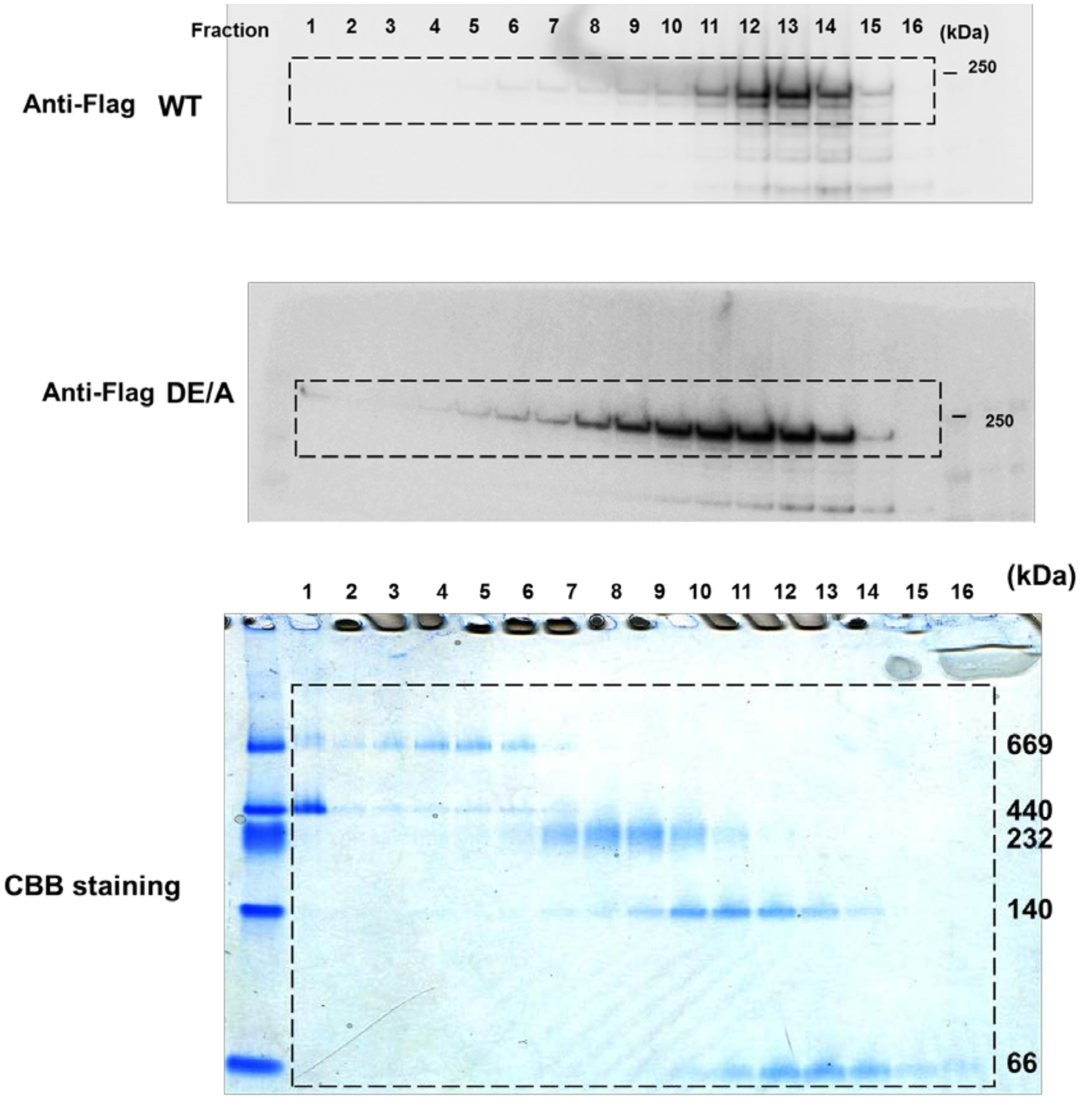

Supplementary Figure 23. Uncropped scans of the blots.
Uncropped scans of Supplementary Figure 6.

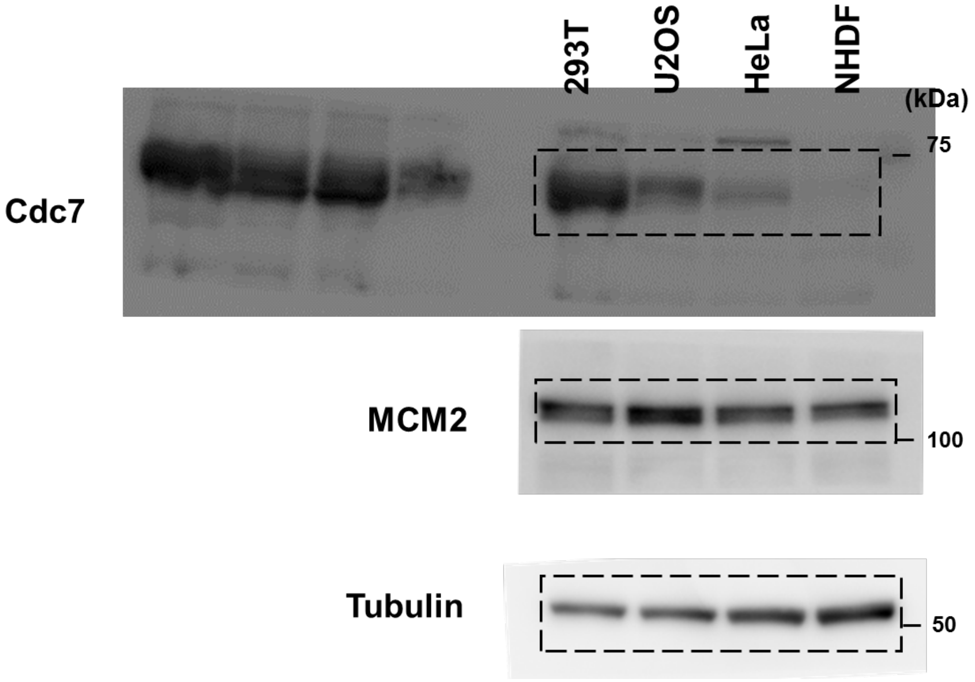

Supplementary Figure 24. Uncropped scans of the blots.
Uncropped scans of Supplementary Figure 7.

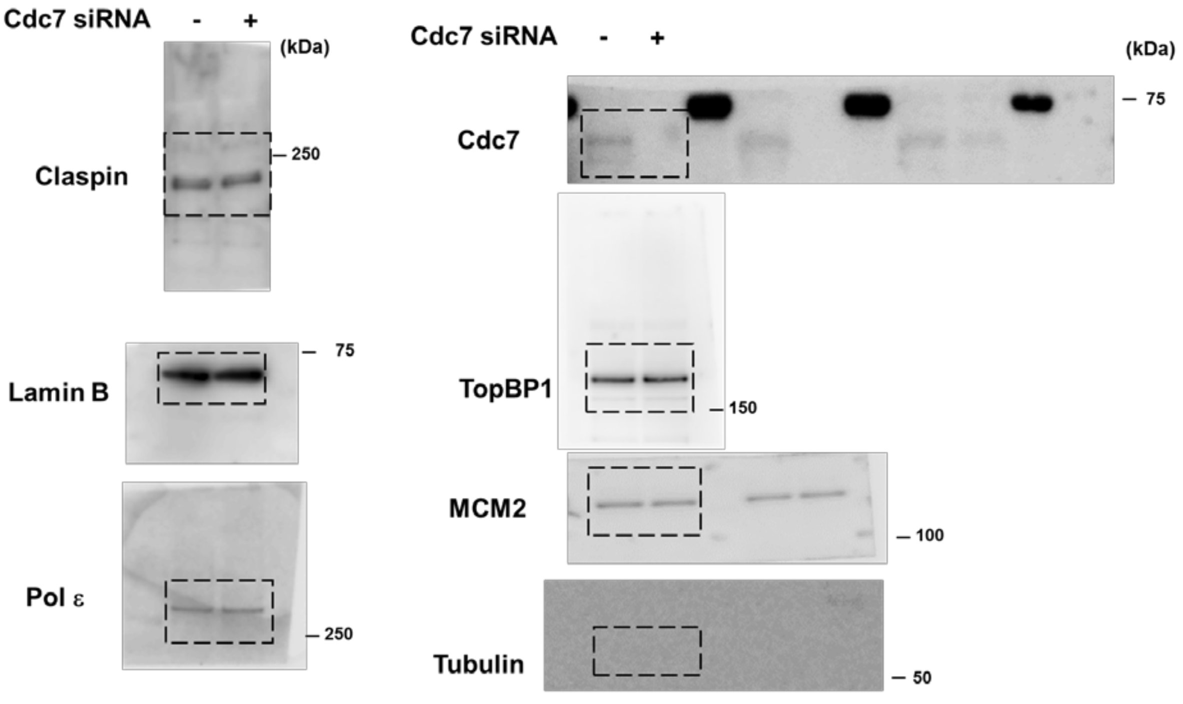

Supplementary Figure 25. Uncropped scans of the blots.
Uncropped scans of Supplementary Figure 8.

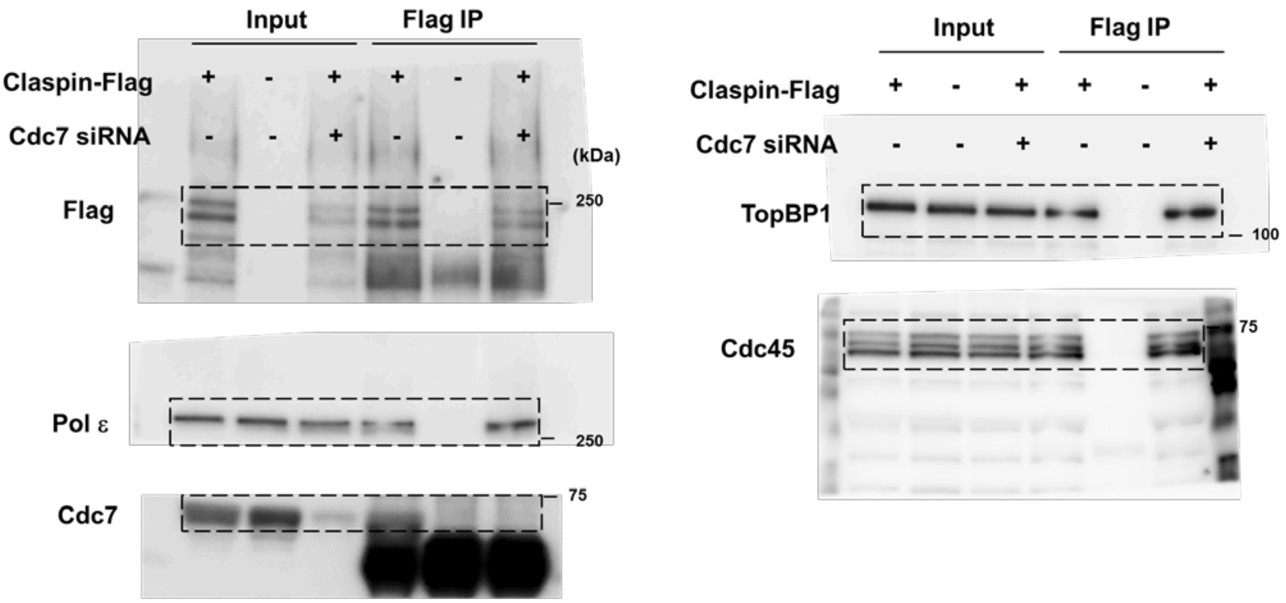

Supplementary Figure 26. Uncropped scans of the blots.
Uncropped scans of Supplementary Figure 9.

a

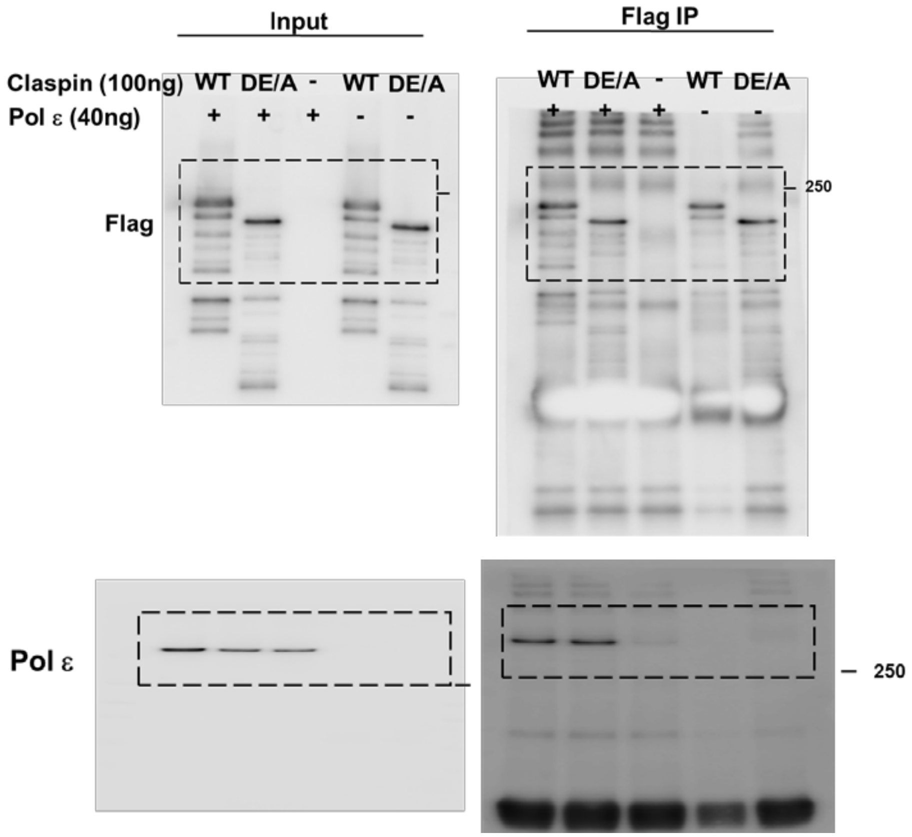

b

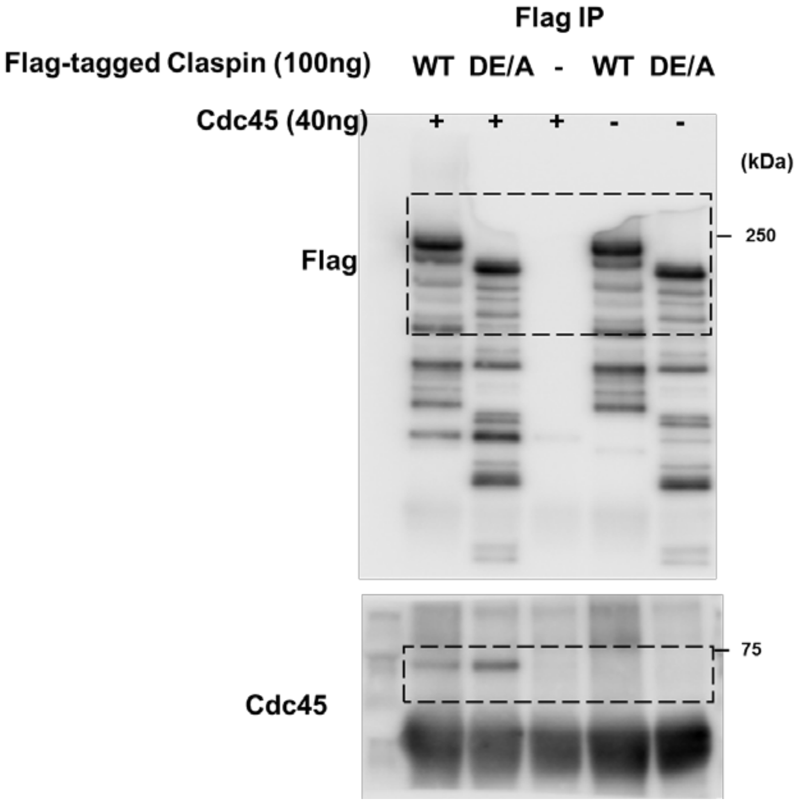

Supplementary Figure 27. Uncropped scans of the blots.
(a)Uncropped scans of Supplementary Figure 10a.(b) Uncropped scans of Supplementary Figure 10b.

SUPPLEMENTARY TABLE 1.

Sequence of the oligonucleotides used in this study

Name	Sequence (5'-3')
**Primers**	
FL-F	ccgctcgagactagtatgacaggcgaggtgggttctg
FL-R	ctagtctagagctctccaaatatttgaagatgc
#2-R	ctagtctagaagtgtctatgatttctttgtg
#9-F	ccgctcgaggcaaatactactgaaatgaa
#9-R	ctagtctagacaatggcaatcgaggcttcaaag
#13-F	ccgctcgagactagtgccagtatggatgagaatgcc
#13-R	ctagtctagacagtatcataaactgactgtcc
#14-R	ctagtctagaaaaagagcctgagcaaagagcaag
#27-R	acctctagactttcttgatttgactctgcagttcc
#27N-F	accggctagcgccagtatggatgagaatgcc
#27N-R	accggctagctttcttgatttgactctgcagttcc
Cdel1-1-R	ccgggatccaaaagagcctgagcaaagag
Cdel1-2-F	ccgggatccgccaagaaagttacagccaaa
Cdel2-1-R	ccgggatcctttcttgatttgactctgcagttcc
Cdel2-2-F	ccgggatccgccaagaaagttacagccaaa
Cdel4-1-R	ccgggatccaaaagagcctgagcaaagag
Cdel4-2-F	ccgggatcccccacagacaaggaagagga
C-del6-1-R	ccggctagcaaaagagcctgagcaaagag
Cdel6-2-F	ccggctagcatacacatgaaaactatgttggatg
Cdel7-1-R	ccgggatccaaaagagcctgagcaaagagcaag
Cdel7-2-F	ccgggatccatacacatgaaaactatgttggatg
Cdel6N-1-1-R	accggctagccttcctgggtagatgtttttca
Cdel6N-2-1-R	accggctagcctctgatgaggctggagtgga
Cdel6N-3in-F	accggctagccaggctgaaaaacatctacc
Cdel6N-4in-F	accggctagccaggatgcctccactccag
Cdel6N-5in-F	accggctagcgacaaggaagaggaagacga
#2 PIP-1-F	gctaaaaccgctcatgatgccgccaaacgtaaac
#2 PIP-2-R	ggcggcatcatgagcggttttagcctcaggcata

Genotyping primers

Cg 1F	aaacccgaaaaaccaagcgaatctg
Cg 2R	aaaacccgaaaaaccaagcgaatc
Cg 1Mr	agtgtggggacatcagctgca

Claspin knockdown

Sense	uuggccacugauuucaauutt
anti-sense	aauugaaaucaguggccaatt

Y-fork oligonucleotides

dT32-32mer

tttttttttttttttttttttttttttttttttggttggccgatcaagtgcccagtcacgacgtt

32mer-dT32

aacgtcgtgactgggcacttgatcggccaacctttttttttttttttttttttttttttttttt

REFERENCES

1. Yamazaki, S. *et al.* Rif1 regulates the replication timing domains on the human genome. *EMBO J.* **31,** 3667–3677 (2012).
